# Modulating the Inclusive and Coordinating Ability of Thiacalix[4]arene and Its Antenna Effect on Yb^3+^-Luminescence via Upper-Rim Substitution

**DOI:** 10.3390/molecules27206793

**Published:** 2022-10-11

**Authors:** Sergey N. Podyachev, Svetlana N. Sudakova, Rustem R. Zairov, Victor V. Syakaev, Alexey N. Masliy, Michal Dusek, Aidar T. Gubaidullin, Alexey P. Dovzhenko, Daina N. Buzyurova, Dmitry V. Lapaev, Gulnaz Sh. Mambetova, Vasily M. Babaev, Andrey M. Kuznetsov, Asiya R. Mustafina

**Affiliations:** 1 Arbuzov Institute of Organic and Physical Chemistry, FRC Kazan Scientific Center of RAS, Arbuzov Str. 8, 420088 Kazan, Russia; 2 Department of Inorganic Chemistry, Kazan National Research Technological University, K. Marx Str. 68, 420015 Kazan, Russia; 3 Institute of Physics of the Czech Academy of Sciences, Na Slovance 2, 182-21 Prague, Czech Republic; 4 Department of Physical Chemistry, Kazan (Volga Region) Federal University, Kremlyovskaya Str. 18, 420008 Kazan, Russia; 5 Zavoisky Physical-Technical Institute, FRC Kazan Scientific Center of RAS, Sibirsky Tract 10/7, 420029 Kazan, Russia

**Keywords:** calix[4]arenes, Yb^3+^ complexes, luminescence, halogen-bonding, X-ray analysis

## Abstract

The present work introduces the series of thiacalix[4]arenes (H_4_L) bearing different upper-rim substituents (R = H, Br, NO_2_) for rational design of ligands providing an antenna-effect on the NIR Yb^3+^-centered luminescence of their Yb^3+^ complexes. The unusual inclusive self-assembly of H_3_L^−^ (Br) through Br…π interactions is revealed through single-crystal XRD analysis. Thermodynamically favorable formation of dimeric complexes [2Yb^3+^:2HL^3−^] leads to efficient sensitizing of the Yb^3+^ luminescence for H_4_L (Br, NO_2_), while poor sensitizing is observed for ligand H_4_L (H). X-ray analysis of the single crystal separated from the basified DMF solutions of YbCl_3_ and H_4_L(NO_2_) has revealed the transformation of the dimeric complexes into [4Yb^3+^:2L^4−^] ones with a cubane-like cluster structure. The luminescence characteristics of the complexes in the solutions reveal the peculiar antenna effect of H_4_L(R = NO_2_), where the triplet level at 567 nm (17,637 cm^−1^) arisen from ILCT provides efficient sensitizing of the Yb^3+^ luminescence.

## 1. Introduction

Calix[n]arenes and their thia-analogues continue to excite interest as a promising basis for design and synthesis of lanthanide complexes, which were successfully applied in developing nanosensors and contrasting agents [1,2]. The main advantage of a cyclophanic backbone is the feasibility of the structural diversifications, which allows embedding of different groups, in turn allowing either complex ability of the calixarene derivatives or tuning their acid/base and complexing properties through electronic effects of the substituents [3,4]. Moreover, the presence of the cyclophanic cavity results in unique inclusive complex formation mainly driven by the electron-donating ability of the cavity [5,6]. The present work is focused on upper-rim substituted thiacalix[4]arenes since preorganization of the four phenolic moieties provides excellent chelating properties towards lanthanide ions, followed by ligand-to-lanthanide energy transfer [1,7,8,9]. It is worth noting that upper-rim substitution of thiacalix[4]arenes was already documented as the tool to both increase their water solubility [9,10] and modify their sensitizing effect on lanthanide-centered luminescence [1,7,8,9]. In particular, embedding of the bromine-substituents onto the upper rim of thiacalix[4]arenes allows to modify the ligand-centered triplet level responsible for feeding of the excited lanthanide-centered levels [8]. Incorporation of nitro-groups onto the upper rim of thiacalix[4]arene has also been reported [11,12,13,14].

Ytterbium compounds exhibiting near-infrared (NIR) luminescence are widely applied building blocks of nanomaterials for bioimaging and photothermal therapy [15,16,17,18,19,20,21,22]. This is due to the fact that Yb^3+^-centered luminescence exhibits the greatest intensity among other NIR-emitting lanthanide ions [23], which, in turn, derives from the large energy gap, 10,250 cm^−1^, between its emitting level and the ground state [24]. The poor feeding of the excited ^2^F_5/2_-level of Yb^3+^ due to forbiddance of *f*-*f* transitions raises a question of their feeding through ligand-to-metal [25,26] or metal-to-metal energy transfer and minimizing of radiationless transitions from the excited Yb^3+^-level to lower laying vibrational levels of ligands [27,28,29]. The reports of Iki et al. [30,31,32] highlight the advantage of the thiacalix[4]arene backbone in developing NIR-luminescent Yb^3+^ complexes due to the specific rigid inner-sphere environment resulting from the sandwich-like coordination of the Yb^3+^ ions between two phenolate rims of the thiacalix[4]arene derivatives.

However, an impact of the upper-rim substitution of thiacalix[4]arenes by the bromine and nitro-groups on the developing of bright NIR Yb^3+^-centered luminescence has not been highlighted. A combination of electron-withdrawing (NO_2_) and electron-donating moieties (OH, O^−^) in thiacalix[4]arene molecules should produce an intraligand charge transfer (ILCT) absorption band in the visible range similar to that in the electronic spectra of nitrophenolates [33]. Literature data demonstrate fine examples of convenient excitation of Yb^3+^ NIR luminescence by means of an ILCT absorption band in the visible range [25,26,34]. Thus, it is worth assuming that combination of the rigid inner-sphere ligand environment of Yb^3+^ ions with excitation of an Yb^3+^-centered luminescence by means of an ILCT absorption band can be a tool to develop bright Yb^3+^-centered luminescence.

**Figure 1:**
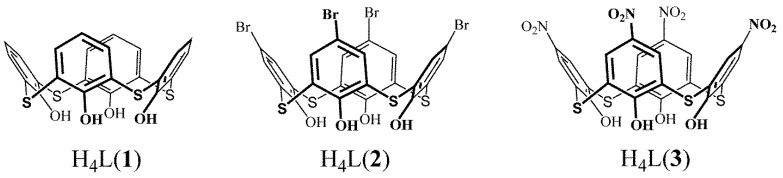
Thiacalix[4]arenes H_4_L(**1**–**3**) studied in this work.

The present work represents thiacalix[4]arenes H4L(**1**–**3**) (Figure 1) with different upper-rim substituents (R = H, Br and NO_2_). The structural variation in the upper-rim substituents is aimed to highlight their impact on producing unique supramolecular structures, in turn derived from inclusive or coordinating abilities of the bromo- and nitro-substituted thiacalix[4]arenes. Such structure variation is also aimed at distinguishing different structure effects on Yb^3+^-centered luminescence of the corresponding complexes, including: (1) structure rigidity effect derived from bulky substituents (Br, NO_2_); (2) interaction of the lone pairs of NO_2_ group with π * orbitals of the aromatic ring, which is known to quench Eu^3+^ and Tb^3+^ luminescence through shortening of the triplet excited state lifetime [35]; (3) participation of the triplet level arisen from the ILCT in the feeding of the low-energy excited states of Yb^3+^.

The unique supramolecular structures of bromo-substituted thiacalix[4]arene and the ytterbium complex of nitro-substituted thiacalix[4]arene determined by single-crystal X-ray diffraction (XRD) data will be discussed in correlation with the literature data and experimental results on complexation of Yb^3+^ with thiacalix[4]arenes in DMF solutions. The coordination modes of Yb^3+^ in the complexes will be revealed by computational modelling. Steady state and time-resolved Yb^3+^-centered luminescence will be correlated with both spectral properties and structural features of the ligands in order to recognize the impact of different factors, including the ILCT, on the luminescence of the complexes.

## 2. Results and Discussion

### 2.1. UV–Vis Absorption Behavior of H_4_L(**3**) and Crystal Structure of H_3_L(**2**)^−^

Discussion of the complex ability and antenna-effect of H_4_L(**1**–**3**) ligands in basified DMF solutions should be preceded by analysis of their acid–base behavior revealed through their spectral behavior at different concentrations of TEA. Both spectral and acid–base behaviors of H_4_L(**1**,**2**) in DMF solutions have already been published [7,8], and a correlation between deprotonation and spectral changes for H_4_L(**3**) was reported in aqueous solutions [12,13].

**Figure 2:**
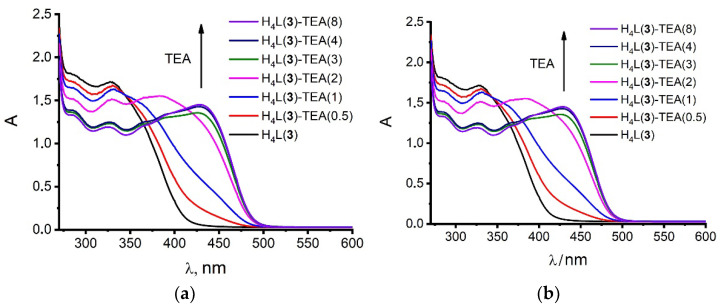
(**a**) UV–Vis spectra and (**b**) the absorbance values (A) (λ = 430 nm) in neutral and basified DMF solutions of H_4_L(**3**) (C_H4L_ = 0.05 mM) at the varied TEA/L (0–8) molar ratio.

The electronic absorption spectra of **3** recorded in the neutral and basified DMF solutions are represented in Figure 2a. The enhanced electronic absorbance at 340–400 nm revealed from the spectrum of **3** in the neutral DMF solution (Figure 2a) is explained by the enhanced first step deprotonation of H_4_L(**3**) since similar spectral behavior of H_4_L(**3**) in the aqueous solutions was correlated with pK_1_ = 2.75 [13]. The enhanced acidity of H_4_L(**3**) derives from both its cyclophanic structure and the electron-withdrawing effect of *p*-nitro-substituents, which differentiates H_4_L(**3**) from H_4_L(**1**,**2**).

The low-energy band of H_4_L(**3**) is both red-shifted and more intensive (Figure 2a) than the shoulder at 340 nm for H_4_L(**2**) [8], which, in turn, is more pronounced than that of H_4_L(**1**) [8]. The spectral behavior of H_4_L(**3**) in the basified DMF solutions is characterized by the appearance of electronic absorbance at ~450–500 nm (Figure 2a). The red-shifting of the low-energy band of H_4_L(**3**) versus those of H_4_L(**1**,**2**) derives from the well-known high electron-withdrawing effect of nitro-substituents, resulting in the appearance of the ILCT absorption band. It is worth noting that the electronic absorption of H_4_L(**3**) in the basified DMF solutions is in the longer wavelengths range, ~450–500 nm compared to the absorption of *p*-nitrophenol (~400–450 nm) in the alkaline solutions [33]. Thus, the cyclophanic structure of H_4_L(**3**) favors lower energy transitions, along with the effect of the *p*-nitro-substituents, which is the reason for the peculiar spectral behavior of H_4_L versus that of *p*-nitrophenol. As is evident from the titration plot in Figure 2b, two-step deprotonation of H_4_L(**3**) is realized in the basified DMF solutions, while only one-step deprotonation was reported for H_4_L(**1**,**2**) [7,8].

**Figure 3:**
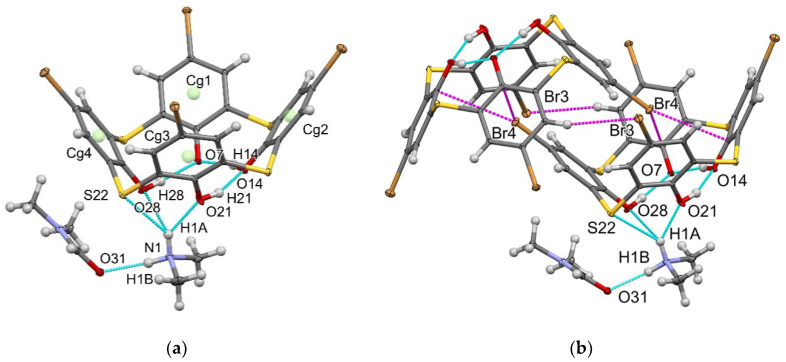
(**a**) O-H…O, N-H…O and N-H…S hydrogen bonds (blue dashed lines) (**a**) and Br…π; (**b**) C-H…Br interactions (magenta dashed lines) in the crystal H_3_L(**2**)^−^·(CH_3_)_2_NH_2_^+^·DMF.

The above-mentioned deprotonation of the phenolic rims of H_4_L(**1**–**3**) facilitates the electron-donating ability of their cavities, which promotes unique intermolecular inclusive interactions and coordination of metal ions. These interactions are clearly demonstrated by XRD analysis of the single crystals (H_3_L(**2**)^−^·(CH_3_)_2_NH_2_^+^·DMF) grown from the DMF solutions of **2** basified by dimethylamine (Figure 3). The thiacalixarene molecule in an almost perfect *cone* conformation with close values of dihedral angles of opposite aromatic rings is located in the general position of the triclinic unit cell (Figure 3). However, the molecule loses its own C_4_ symmetry due to its transformation into salt form, interaction with the solvent molecule (Figure 3a) and specific inclusive interactions (Figure 3b). The crystal structure data (Table S1) and the parameters of the intra- and intermolecular interactions (Tables S2 and S3 and Figures S1–S4) are represented in the Supplementary Materials.

An interesting feature of the intermolecular interaction revealed in single crystals is formation of peculiar centrosymmetric dimers of thiacalix[4]arenes (Figure 3b). Substituent Br3 participates in the strong C-H…Br interaction with the hydrogen H3 of the phenyl ring of the neighboring molecule; the H3…Br3 distance is 3.03 Å. Pairwise incorporation of the bromine substituents designated as Br4 of one molecule into the cavity of another can be stabilized by four Br…π contacts with the four phenyl rings of the thiacalix[4]arene. However, in accordance with the IUPAC criteria [36], only one (Cg2(C9 ÷ C14)…Br4) of the four contacts can be called a halogen bond since the rest of them do not follow the rule of directionality of such bonds. Moreover, two of them have distances between the centers of the bromine and the nearest carbon atoms slightly more than the sum of their van der Waals radii (see Table S3 in Supplementary Materials). However, the formal criteria for such interactions used in the PLATON program [37] allows to consider all the contacts to the Br…π type (for the contact parameters, see Table S3).

It is worth noting the diversity of the Br…π contacts: in particular, the C-Br bond is directed to carbon C14 (Cg2(C9 ÷ C14)…Br4), while, in the contact Cg1(C2 ÷ C7)…Br4, the bromine atom is in an intermediate position between the center of the aromatic cycle and atom C7. In the case of contacts Cg3(C16 ÷ C21)…Br4 and Cg4(C23 ÷ C28)…Br4, the bromine atom is closer to the centers of aromatic rings, which are electron-deficient regions. The aforesaid provides one more example of the unique ability of the bromine substituents to interact with both nucleophilic and electrophilic centers, which has gained great attention in the last decade [38].

The revealed short Br…π contacts are predominantly driven by polarized electrostatic attractions between the electron-deficient bromo-substituents and the electron donating cavity of H_3_L^−^(**2**) as a Lewis base [39,40]. It is worth noting that halogen bonds have already been highlighted as the driving force of inclusion of halogen-substituted benzenes into the cavities of calix[4]arene derivatives [41]. A search of the Cambridge Structural Database (CSD version 5.43, March 2022 release) for all structures containing upper-rim halogen substituted thia- and calix[4]arenes (68 hits) reveals the only example of dimeric or cog-like self-inclusion of distal substituted dibromocalix[4]arene bearing the two propoxy groups on the lower-rim [42]. This indicates that the present inclusive self-assembly based on halogen bonds is rather rare. Moreover, dimeric self-assembly is also stabilized by the C-H…Br interaction driven by the electron donating capacity of the bromo-substituents (Figure 3b and Figure S1). Thus, the Janus-like nature of the bromine substituents provides additional interactions, which, along with S…π and π… π interactions, form the one-dimensional supramolecular motif (Figure S1). Such chains are bound in a perpendicular direction due to Br…π and C-H…Br contacts (Figure S2), forming a two-dimensional supramolecular motif—a layer of thiacalixarene dimeric fragments, where the dimeric structure shown in Figure 3b serves as a supramolecular synthon. Translation of such synthon in three directions forms a crystal packing as a whole (Figures S3 and S4). It is worth noting that the supramolecular packing is characterized by a sufficiently high packing factor of 0.717, which is closer to the upper limit of the packing factor values for crystals of organic compounds (0.65–0.75).

### 2.2. Complex Formation of H_4_L(**1–3**) with Yb^3+^ Ions

Compounds H_4_L(**1**,**2**), previously represented as efficient ligands for Tb^3+^ ions, provide their tight coordination followed by efficient sensitizing of terbium-centered luminescence [7,8]. Discussion of the complex formation of ligand H_4_L(**3**) in solutions is worth preceding by a presentation of the structure determined by XRD analysis of the single crystals grown from basified DMF solution containing H_4_L(**3**) and Yb^3+^ ions in a 1:1 molar ratio.

The single crystals suitable for XRD analysis were grown through several months staying of the basified DMF solutions of YbCl_3_ and H_4_L(**3**) mixed in a 1:1 ratio, while no single crystals appeared in the same conditions for ligands H_4_L(**1**,**2**). Moreover, in the case of the Yb(NO_3_)_3_, we could not succeed in obtaining any crystals.

**Figure 4:**
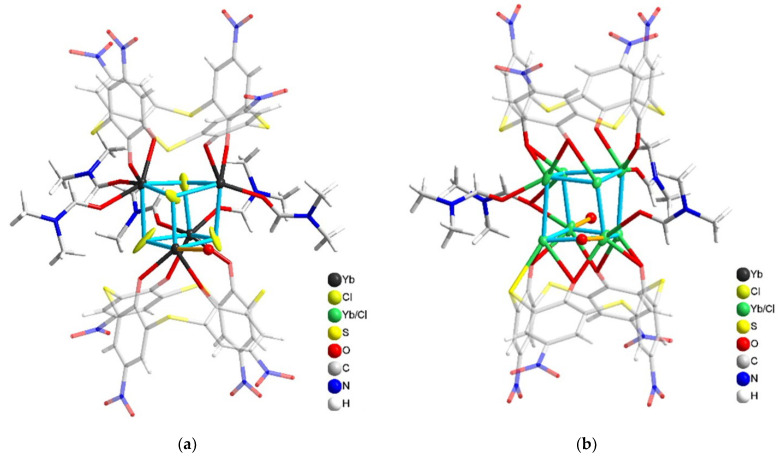
Ligands coordinated to the Yb_4_Cl_4_ “cube”: (**a**) with separated Yb and Cl positions. Cl atoms are disordered and not bonded to ligands; (**b**) with mixed Yb and Cl positions. L^4−^ anions are plotted semi-transparent. A water molecule is represented by an oxygen atom plotted as a red ball.

X-ray analysis of the separated single crystal revealed the large and strongly disordered structure in the monoclinic P21/n space group. A detailed description of the crystal structure data is in Supplementary Materials (Figures S5 and S6 and Table S4). The cell unit consists of two individual complexes in its composition with 2:1 (Yb:L) stoichiometry, although, as has been aforesaid, YbCl_3_ and H_4_L(**3**) were mixed in a 1:1 ratio (Figure 4). Both of them contain a rather specific dimeric cubane-like structure of complex. The cluster coordination of four Yb^3+^ ions with eight phenolates of two completely deprotonated *p*-nitro-thiacalix[4]arene anions (L^4−^) is stabilized by the bridge-like coordination of chloride ions with the coordination number of Yb^3+^ ions equal to 6 and 7, according to Figure 4. Such values are rather scarce in comparison with coordination numbers 8 and 9 predominantly reported in the literature [43]. However, the data quality is insufficient to reveal such structural details as a location and number of water molecules supporting the structure.

Stabilization of transition metal ions clusters through coordination by thiacalix[4]arenes is well-represented by the review [44]. However, the cubane-like lanthanide coordination (Ln = Gd^3+^, Eu^3+^ and Tb^3+^) has been found only for sulfonylcalix[4]arene, where the cluster motif is supported by coordination of the lanthanide ions via both phenolates and sulfonyl oxygen atoms along with four bridging acetate ligands [44,45].

Both stoichiometry and structure of the cubane-like dimeric complex of Yb^3+^ with ligand H_4_L(**3**) significantly differ from those of the dimeric terbium complexes formed by ligands **1** and **2** in the DMF solutions [7,8]. Thus, the revealed dimeric structure may either derive from the complex formation mode in solutions or be mainly affected by the crystal packing forces. However, the lanthanide contraction may be one more reason for specificity in the coordinative behavior of Yb^3+^ versus its counterparts from the middle of the lanthanide series. Therefore, the complex formation of H_4_L(**3**) with Yb^3+^ ions will be represented along with that of H_4_L(**1**,**2**) in the DMF solutions.

**Figure 5:**
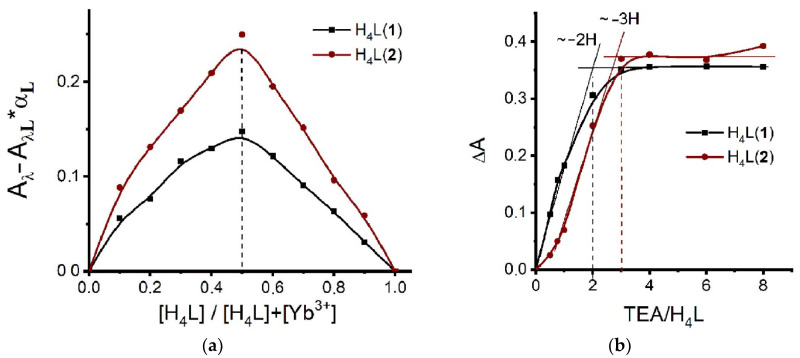
The Job’s plot profiles of DMF solutions at the varied H_4_L:Yb^3+^ molar ratios: (**a**) H_4_L(**1**) (λ = 340 nm), H_4_L(**2**) (λ = 350 nm) ([H_4_L] + [Yb^3+^] += 0.1 mM, L:TEA (1:8)); (**b**) ΔA of the DMF solutions of H_4_L with Yb(NO_3_)_3_ at the varied TEA:L molar ratios: H_4_L(**1**) (λ = 340 nm), H_4_L(**2**) (λ = 350 nm). C_Yb_3+ = C_H4L_ = 0.1 mM.

The intraligand electronic absorbance of H_4_L(**1**–**3**) is a convenient tool to reveal and compare their complex formation abilities towards lanthanide ions. The UV–Vis spectral data calculated and represented in the form of the Job’s plots in Figure 5a demonstrate no specificity of Yb^3+^ complex formation with ligands H_4_L(**1**) and H_4_L(**2**) in comparison with earlier obtained data for their Tb^3+^ complexes [7,8]. The complex formation of Yb^3+^ is accompanied by the deprotonation of two and three protons under their complex formation with H_4_L(**1**) and H_4_L(**2**) (Figure 5b), which is also in good agreement with the terbium complex formation.

**Figure 6:**
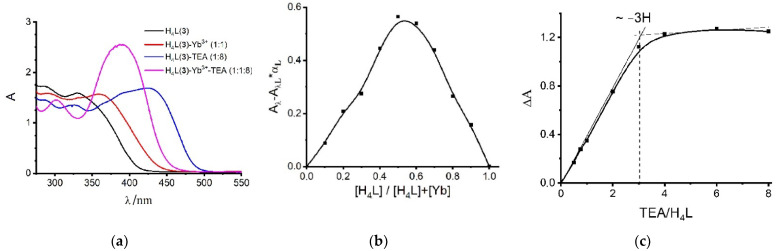
(**a**) UV-Vis absorption spectra of H_4_L(**3**) (C_L_ = 0.05 mM) in DMF; H_4_L(**3**) with Yb(NO_3_)_3_ (C_Yb_^3+^ = 0.05 mM) (H_4_L(**3**)-Yb^3+^ (1:1)); H_4_L(**3**) with TEA (C_TEA_ = 0.4 mM) (H_4_L(**3**)-TEA (1:8)); H_4_L(**3**) with Yb(NO_3_)_3_ (C_Yb_^3+^ = 0.05 mM) and TEA (C_TEA_ = 0.4 mM) (H_4_L(**3**)-Yb^3+^-TEA (1:1:8)); (**b**) the Job’s plot profiles of DMF solutions at the varied H_4_L(**3**):Yb^3+^ molar ratios: (λ = 400 nm) ([Yb^3+^] + [H_4_L] = 0.05 mM, [H_4_L]:TEA (1:8)); (**c**) ΔA of the DMF solutions of H_4_L(**3**) with Yb(NO_3_)_3_ at the varied TEA:L molar ratio (λ = 400 nm, C_Yb_^3+^ = C_L_ = 0.05 mM).

It should be noted that similar data for *p*-nitro-thiacalix[4]arene H_4_L(**3**) have not been reported. The addition of Yb^3+^ to the basified solution of H_4_L(**3**) results in increased absorbance at 400 nm with the disappearance of the lower energy absorption bands at 450–500 nm (Figure 6a). Such spectral behavior provides a clear indication of Yb^3+^ coordination via the lower phenolic rim of H_4_L as the reason for restricted charge transfer from phenolate to nitro-groups. The quantitative analysis of the spectral changes resulting from the concentration variation in both H_4_L(**3**) and Yb(NO_3_)_3_ through the Job-plotting (Figure 6b) indicates that the complex formation of Yb^3+^ ions with H_4_L(**3**) in the basified DMF solutions predominantly occurs in 1:1 stoichiometry. However, similar with H_4_L(**1**, **2**), the non-symmetrical shape of the Job plot (Figure 5a and Figure 6b) indicates that the 1:1 stoichiometry is contributed by the complex forms with 2:1 (Yb:L) stoichiometry. It is worth noting that the Job plots are indistinguishable in the solutions of Yb(NO_3_)_3_ and YbCl_3_, which points to predominance of the 1:1 stoichiometry in the recently prepared basified DMF solutions (Figure S7).

Thus, similar to the other thiacalix[4]arenes H_4_L(**1**,**2**), the 1:1 complex stoichiometry is predominant in the complex formation of H_4_L(**3**) with Yb^3+^, followed by deprotonation of three phenolic moieties (Figure 6c). It is interesting that longer storage of the solutions with YbCl_3_ resulted in obtaining crystals having the 2:1 stoichiometry. In accordance with Le Chatelier’s principle, the phase separation of the crystals is the main driving force for both further deprotonation of the ligand and transformation of the complex stoichiometry from 1:1 to 2:1. It is also worth noting that the DMF molecule caps the cyclophanic cavities, and it should be considered as one more factor for stabilizing the structure.

### 2.3. Diffusion NMR Spectroscopy

The NMR spectral changes of ligands H_4_L(**1**–**3**) resulted from their complex formation in alkalized DMSO-*d*^6^ solutions were analyzed for diamagnetic Lu^3+^ ions in order to exclude the broadening of signals due to the paramagnetic effect of Yb^3+^ ions. The interference of the signals arising from the different complex forms restricts the correct evaluation of self-diffusion coefficients in the case of H_4_L(**1**). Thus, the self-diffusion coefficients were obtained for the complexes with H_4_L(**2**,**3**) (Table 1). Their quantitative analysis allows estimating that the self-diffusion coefficient of ligand H_4_L(**2**) decreases by 16% under the complex formation with Lu^3+^ (Table 1), while in the case of La^3+^_,_ a more significant decrease (21%) was reported [8]. The self-diffusion coefficient for the thiacalix[4]arene H4L(3) under the complex formation with Lu^3+^ becomes lower by 14% (Table 1). According to the literature data, the decrease of *D*_s_ by ~25% testifies to the dimerization of the molecules in the solutions [46,47,48]. Therefore, the less pronounced decrease in self-diffusion coefficients *D*_s_ of ligands H_4_L(**2**,**3**) under the complex formation with Lu^3+^ versus La^3+^ ions indicates the greater contribution of the monomeric forms. In particular, the accumulation of dimeric complex forms is ~65% and 55% for ligands H_4_L(**2**) and H_4_L(**3**), correspondingly.

### 2.4. MALDI-TOF Mass Spectrometry Data

**Figure 7:**
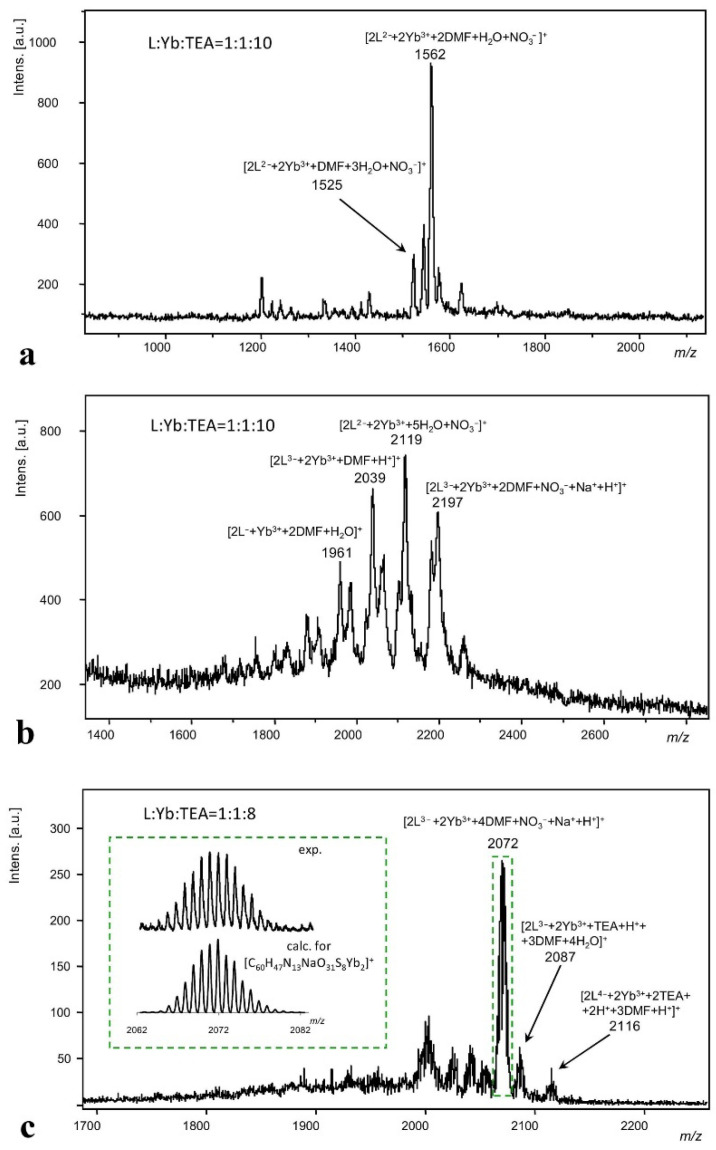
MALDI-TOF mass spectra of the H_4_L-Yb^3+^-TEA (1:1:8, 1:1:10) system: L = (**a**) H_4_L(**1**); (**b**) H_4_L(**2**); (**c**) H_4_L(**3**).

MALDI-TOF mass spectra were recorded for the mixtures Yb^3+^: L: TEA (1:1:8, 1:1:10) in DMF solutions with registration of positively charged ions (Figure 7). The intensive peaks at *m/z* = 1500–1600 assignable to the dimeric (2:2) complexes ([2L^2−^ + 2Yb^3+^ + DMF + 3H_2_O + NO_3_^−^]^+^, [2L^2−^ + 2Yb^3+^ + 2DMF + H_2_O + NO_3_^−^]^+^) are revealed for ligand **1** (Figure 7a). The intensive peaks at *m/z* 1900–2200 assignable to the monomeric ([2L^−^ + Yb^3+^ + 2DMF + H_2_O]^+^) and dimeric complex forms ([2L^2−^ + 2Yb^3+^ + DMF + H^+^]^+^, [2L^2−^ + 2Yb^3+^ + DMF + H_2_O + NO_3_^−^]^+^, [2L^3−^ + 2Yb^3+^ + 2DMF + NO_3_^−^ + Na^+^ + H]^+^) (Figure 7b) are observed for ligand **2**. In turn, only the peaks at *m/z* 1950–2150 ([2L^3−^ + 2Yb^3+^ + 4DMF + NO_3_^−^ + Na^+^ + H^+^]^+^, [2L^3−^ + 2Yb^3+^ + 3DMF + 4H_2_O + TEA + H^+^]^+^) (Figure 7c) assignable to dimeric complexes are registered in the case of ligand **3**. Thus, the MALDI-TOF mass spectra confirm the tendency of ligands H_4_L(**1**–**3**) to form dimeric complexes with Yb^3+^.

### 2.5. Computational Modeling of the Yb^3+^Complexes with p-Nitrothiacalix[4]arene (H_4_L(**3**))

The DFT calculations were successfully applied in recognition of the impact of the complex stoichiometry and structure on its stability for the lanthanide complexes with the calix[4]arene and thiacalix[4]arene derivatives, including, in particular, the complexes with ligands H_4_L(**1**) and H_4_L(**2**) [7,8]. The DFT calculations of ytterbium complexes with ligand **3** are based on the previously reported thermodynamically favorable structures of Tb^3+^ complexes with ligands H_4_L(**1**) and H_4_L(**2**) with the assumption of the terbium coordination number (CN) being equal to 8. The CN-value of Yb^3+^ can be either 8 or 7 in accordance with the well-known “lanthanide contraction effect”. However, the literature data [49] reveal relatively small differences in the ionic radii of Yb^3+^ (1.125 Å) and Tb^3+^ (1.180 Å) ions. This, in turn, argues for the realization of CN = 8 for the Yb^3+^ complexes with H_4_L(**3**) in the solutions [50].

**Figure 8:**
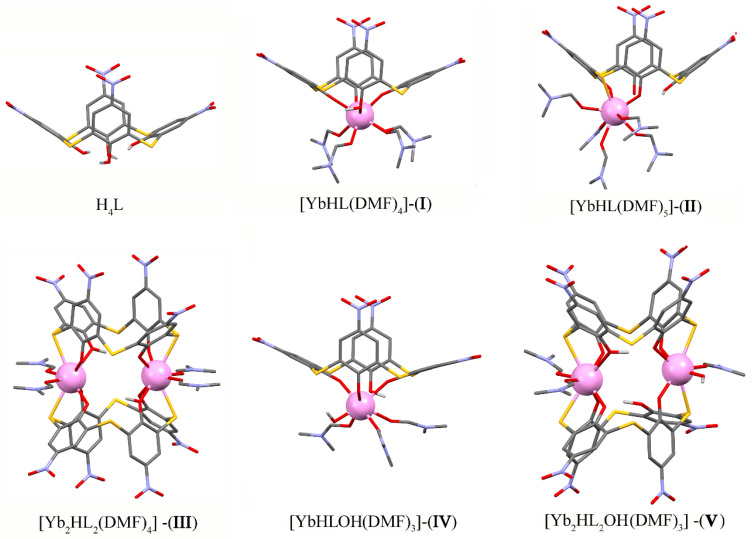
DFT-optimized structures of ligand H_4_L(**3**) and its Yb^3+^ complexes. Only hydrogen atoms of phenolic groups are shown for clarity.

The diversity of the complex formation modes is represented by both monomeric (1:1) and dimeric (2:2) complex forms. The 1:1 complex formation leading to [YbHL] can derive from the coordination of Yb^3+^ via either four oxygens of phenolic/phenolate lower rim of HL^3−^(**3**) ([YbHL(DMF)_4_]-(**I**) in Figure 8) or via two oxygen and one sulfur atom ([YbHL(DMF)_5_]-(**II**) in Figure 8).

The formation of [YbHL(DMF)_4_]-(**I**) and [YbHL(DMF)_5_]-(**II**) from the aqua complex [Yb(H_2_O)_8_]^3+^ can be designated by Equations (1) and (2), where the Yb^3+^ coordination sphere is saturated by four and five DMF molecules, correspondingly:

[Yb(H_2_O)_8_]^3+^ + H_4_L + 4DMF + 3TEA ⇆ [YbHL(DMF)_4_]-(**I**)+ 8H_2_O + 3HTEA^+^ (1)

[Yb(H_2_O)_8_]^3+^ + H_4_L + 5DMF + 3TEA ⇆ [YbHL(DMF)_5_]-(**II**) + 8H_2_O + 3HTEA^+^ (2)

The complexes (**I**–**II**) can undergo transformation into the 2:2 complex (**III**) (Figure 8) in accordance with Equations (3) and (4):

2[YbHL(DMF)_4_] -(**I**) ⇆ [Yb_2_HL_2_(DMF)_4_] -(**III**) + 4DMF (3)

2[YbHL(DMF)_5_] -(**II**) ⇆ [Yb_2_HL_2_(DMF)_4_] -(**III**) + 6DMF (4)

The thermochemical parameters of complexes **I**, **II** and **III** are collected in Table 2. The ΔG^0^_298_-values of monomeric complexes (**I**,**II**) formation indicate that they are thermodynamically favorable and mainly provided by the enthalpy contribution (Table 2). However, the dimeric complex formation (equilibriums 3, 4) is an entropically driven process, which differentiates it from the formation of monomeric complexes (**I**,**II**). The 2:2 complex formation undergoes coordination of each Yb^3+^ ion via two oxygen and one sulfur atom of H_4_L(**3**), with further saturation of the coordination sphere of Yb^3+^ by two DMF molecules (structure [Yb_2_HL_2_(DMF)_4_]-(**III**) in Figure 8).

Lanthanide contraction may be the reason for specificity in the coordinative behavior of Yb^3+^ or Lu^3+^ versus their counterparts from the beginning and middle of the lanthanide series. The specificity is in the enhanced acidity of the inner-sphere water molecules, in turn resulting in their transformation into hydroxyls. The formation of the hydroxyl-containing complex forms [YbHL(OH)(DMF)_3_]-(**IV**) and [Yb_2_HL_2_(OH)(DMF_3_]^−^-(**V**) in the presence of TEA can be described by the following equilibriums:

[[Yb(H_2_O)_8_]^3+^ + H_4_L + 3DMF + 4TEA ⇆ [YbHL(OH)(DMF)_3_]^−^-(**IV**) + 7H_2_O + 4HTEA^+^ (5)

[YbHL(OH)(DMF_3_]^−^-(**IV**) + [YbHL(DMF)_4_]-(**I**) ⇆ [Yb_2_HL_2_(OH)(DMF)_3_]^−^-(**V**) + 4DMF (6)

In accordance with the ΔG^0^_298_-values (Table 2), the formation of hydroxy-form (**IV**) is less profitable in comparison with complexes (**I**) and (**II**) in the solution. However, the assembly of [YbHL(OH)(DMF)_3_]-(**IV**) and [YbHL(DMF)_4_]-(**I**) into [Yb_2_HL_2_(OH)(DMF)_3_]^−^-(**V**) is both thermodynamically favorable and entropically driven (Table 2). Thus, complex forms **III** and **V** are more thermodynamically favorable than **I**, **II** and **IV**. Nevertheless, the NMR diffusion results (Table 1) reveal that the aforesaid complex forms are in equilibrium with the diverse monomeric complex forms.

### 2.6. Luminescence Spectroscopy

**Figure 9:**
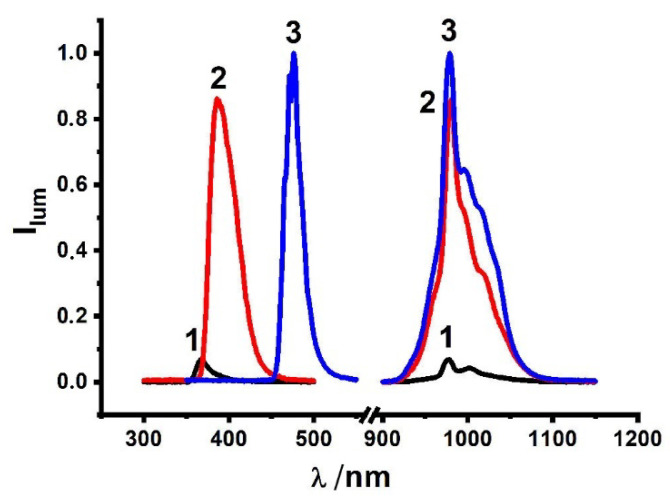
Excitation and emission spectra of Yb^3+^ complexes with H_4_L (**1**–**3**) in the present TEA in DMF solutions.

The complex formation in the solutions is followed by the sensitizing of Yb^3+^-centered luminescence derived from ^2^F_5/2_ → ^2^F_7/2_ transition, with the main emission band centered at 980 nm and the secondary lines at 971, 996, 1025 and 1040 nm arisen from the main crystal field splitting (Figure 9) [16,17,51]. The spectra in Figure 9 demonstrate that the Yb^3+^-centered luminescence is the greatest for the complexes with *p*-nitro- and *p*-bromo-substituted thiacalix[4]arenes H_4_L(**2**,**3**) versus the complex with H_4_L(**1**). The intensity ratios of the bands at 971 and 996 nm, as well as of those at 1025 and 1040 nm, are well-known for their sensitivity to any changes in the inner-sphere ligand environment of Yb^3+^ ions [51]. The ratios at 971 and 996 nm deviate within 1.56–1.72 for the studied ligands, while the ratio of the lower energy luminescence bands (1025 and 1040 nm) is somewhat greater for the complexes with ligand **3** versus those with **1** and **2**, which argues for some peculiarity in the inner-sphere environment of Yb^3+^ in the case of **3**. It is worth noting the equilibration of the above-mentioned 2:2, 1:1 and 2:1 complexes as the factor influencing the inner-sphere environment of Yb^3+^ ions in the complexes with ligands **2** and **3**. Thus, the aforesaid deviation between the spectral patterns of the complexes and ligands **2** and **3** (Figure 9) can be explained by the different ratios of the dimeric to monomeric complex forms.

The average excited state lifetime (τ_av_) values of Yb^3+^ in the complexes with ligands **2** and **3** are 17.68 µs and 23.58 µs (Table 3). The τ-value of the complex with **1** is significantly shorter; thus, its correct measuring lies out of the present work’s scope since the lowest limit of correct lifetime estimation is around 10 µs for a flash lamp used as the excitation source. Altogether, these facts argue for the small number of solvent molecules in the inner sphere environment of Yb^3+^ ion for H_4_L(**2**,**3**) complexes, pointing to the significant contribution of 2:2 form in contrast to 1:1 form for H_4_L (**1**). The steady state intensities of the complexes correlate with their excited state lifetimes (Table 3), thus indicating that the radiationless losses are significantly lower for the complexes with H_4_L(**2**,**3**) versus those with H_4_L(**1**). It is worth noting that the similarity in τ_av_-values of Yb^3+^ in the complexes with ligands **2** and **3** allows to exclude the significant dissipation of the excitation energy in H_4_L(R = NO_2_) caused by the presence of the nitro-substituents as it was found for the lanthanide complexes with nitrobenzoates [35].

The excitation spectra of the complexes also reveal the difference between the ligands H_4_L(**1**–**3**) (Figure 9). In particular, the maximums of the excitation bands exhibit red shifting, which increases in the following series: H_4_L(**1**) (367 nm) < H_4_L(**2**) (386 nm) < H_4_L(**3**) (476 nm).

Low temperature phosphorescence measurements of [Gd_2_L_2_]^2−^ for H_4_L(**3**) were performed to evaluate the energy of the triplet level at 567 nm (17,637 cm^−1^) (Figure S8) and average lifetimes of the ligand-centered phosphorescence of the Gd^3+^ (<τ> = 753 μs). The value is much lower than the previously reported triplet level energies for the complexes with ligands H_4_L**(1**) and H_4_L(**2**) (417 nm (23,981 cm^−1^) and 458 nm (21,834 cm^−1^), accordingly [8]. The energies of the triplet levels of the ligands increase in the following series: H_4_L(**1**) (417 nm) < H_4_L(**2**) (458 nm) < H_4_L(**3**) (567 nm) (Table 3), which is in good correlation with the above-mentioned red-shifting of the excitation bands.

Visible light excitation is especially important for NIR emitting materials employed in biochemistry and cell biology since living tissues are sensitive to UV irritation [34]. This indicates that the advantage of H_4_L(**3**) versus H_4_L(**2**) is the excitation of the bright NIR-luminescence by the lower energy irradiation. Nevertheless, the energy of the triplet level provides a relatively small impact on the Yb^3+^-luminescence of the complexes with ligands H_4_L(**2**,**3**). This argues for the effect of the upper-rim substituents R = NO_2_, Br on the restricted flexibility of the outer-sphere environment of Yb^3+^ ions versus ligand H_4_L(**1**,R = H) as the main reason for the longer excited state lifetimes (Table 3) and brighter NIR luminescence (Figure 9).

## 3. Materials and Methods

*N,N*-dimethylformamide (DMF) (Acros Organics) was distilled over P_2_O_5_. CDCl_3_ (99.8% isotopic purity) and DMSO-*d*^6^ (99.5% isotopic purity) from Aldrich were used for NMR spectroscopy. Triethylamine (Acros Organics), terbium nitrate (Yb(NO_3_)_3_·5H_2_O) and terbium chloride (YbCl_3_·6H_2_O) (Sigma-Aldrich) were used as commercially received without further purification. The structural formulae of the investigated compounds are shown in Figure 1. Tetrathiacalix[4]arenes **1** [52], **2** [53] and **3** [12] were obtained as described in the literature.

### 3.1. Synthesis of Complex 3 with YbCl_3_

The 2.85 ml of 4.5 mM solution of H_4_L (**3**) in DMF was mixed with 0.135 ml (0.1 M) of solution of ytterbium chloride hexahydrate in DMF. To this mixture, 0.015 ml (7.2 mM) of solution of TEA was added. The resulting solution was stored at room temperature for a few months and resulted in formation of yellowish needle-like crystals, which have been used for X-ray analysis.

### 3.2. Physical Measurements and Methods

Detailed descriptions of physical measurements and methods (electronic absorption, NMR experiments, MALDI-TOF mass spectrometry, crystal structure data, luminescence spectroscopy and quantum-chemical modeling) are presented in Supplementary Materials.

## 4. Conclusions

The present work revealed the impact of the bromo- and nitro-substituents embedded at the upper rim of thiacalix[4]arenes on a supramolecular package in crystals and solution behavior of both tetra-bromo and tetra-nitrothiacalix[4]arenes as well as their ytterbium complexes. It was shown that the upper-rim substituents (R = Br, NO_2_) enhance deprotonation of the phenolic rims and generate the unusual inclusive self-assembly of H_3_L^−^(R = Br), revealed through single-crystal XRD analysis. Similar to non-substituted thiacalix[4]arenes, both tetra-bromo and tetra-nitrothiacalix[4]arenes coordinate Yb^3+^ ions into 1:1, 2:2 and 2:1 (Yb:L) complex forms, but the antenna effect of the thiacalix[4]arene-based ligands on the Yb^3+^-centered luminescence is greatly enhanced by the bromo- and nitro-substituents. Quantum chemical study revealed thermodynamic favorableness of the formation of dimeric 2:2 (Yb:[HL]^−^) complexes with a rigid structure as one of the reasons for efficient sensitizing of the Yb^3+^ luminescence. X-ray analysis of the single crystal separated from the basified DMF solutions of YbCl_3_ and H_4_L (R = NO_2_) revealed transformation of the dimeric complexes into [4Yb^3+^:2L^4−^] ones with a cubane-like cluster structure.

Analysis of the time-resolved luminescence in correlation with the triplet energy levels revealed peculiarity in the antenna effect of H_4_L(R = NO_2_), which is efficient sensitizing of the Yb^3+^ luminescence by the triplet level of the ligand at 588 nm arisen from the ILCT without a significant decrease in the lifetime of the excited state caused by dissipation of an excitation energy in H_4_L(R = NO_2_). However, the tetra-brominated ligand provides similar sensitizing due to the smaller radiationless losses of the ligand environment. Nevertheless, the red-shifting of the excitation wavelengths from 360–380 and 370–420 nm for H_4_L(R = H, Br) to 460–500 nm for H_4_L(R = NO_2_) provides an advantage of H_4_L(R = NO_2_) versus H_4_L(R = H, Br) in further bio-applications.

## Figures and Tables

**Table 1:** Self-diffusion constants, hydrodynamic radii for ligands H_4_L(**2**,**3**) (2.5 mM) in DMSO-*d*^6^ solutions before and after addition of Lu^3+^ (2.5 mM) and TEA (15 mM) at 303 K.

System (Molar Ratio)	Self-Diffusion Coefficients (10^−10^ m^2^s^−1^)	Hydrodynamic Radii *r*_H_ (Å)
**2**	2.32	5.5
**2**-TEA (1:6)	2.31	5.3
**2**- Lu^3+^-TEA (1:1:6)	1.95	6.6
**3**	2.37	5.4
**3**-TEA (1:6)	2.32	5.5
**3**- Lu^3+^-TEA (1:1:6)	2.04	6.3

**Table 2:** The calculated thermochemical parameters (ΔH^0^_298_, ΔS^0^_298_ and ΔG^0^_298_) of formation of complexes **I**–**V** with ligand H_4_L(**3**) in the DMF solutions.

Reaction	Composition	ΔH^0^_298_, kJ	ΔS^0^_298_, J/K	ΔG^0^_298_, kJ
1	[YbHL(DMF)_4_]-(**I**)	−234.1	157.2	−281.0
2	[YbHL(DMF)_5_]-(**II**)	−232.8	61.3	−251.1
3	[Yb_2_HL_2_(DMF)_4_]-(**III**)	41.1	217.3	−23.7
4	[Yb_2_HL_2_(DMF)_4_]-(**III**)	35.9	400.5	−83.4
5	[YbHL(OH)(DMF)_3_]-(**IV**)	−193.3	157.2	−240.1
6	[Yb_2_HL_2_(OH)(DMF)_3_]^−^-(**V**)	−27.0	99.7	−56.7

**Table 3:** Energies of lowest triplet states (*T*_1_) of ligands H_4_L(**1**–**3)** in the 2:2 complexes, average lifetimes of the Yb^3+^-centered luminescence (<τ> ^1^) and ligand-centered phosphorescence of the Gd^3+^ complexes (<τ> ^2^).

H_4_L	Triplet Level, *T*_1_ λ, nm (ν, cm^−1^) at 146 K	<τ> ^1^ (μs) at 298 K	<τ> ^2^ (μs) at 146 K
**1**	417 (23,981) ^3^	-	1437 ^3^
**2**	458 (21,834) ^4^	24	301 ^4^
**3**	567 (17,637)	18	421

^1^ for Yb^3+^ complexes. ^2^ for Gd^3+^ complexes. ^3,4^ the previously reported values [7,8].

## Data Availability

The data presented in this study are available in Supplementary Materials.

## Supplementary Materials

The following supporting information can be downloaded at https://www.mdpi.com/article/10.3390/molecules27206793/s1: Structural formulae of the investigated compounds H_4_L(**1**–**3**); NMR spectroscopy [54]; crystal structure data [55,56,57,58,59,60,61,62,63,64]; the crystal structure data and the parameters of the intra- and intermolecular interactions for H_3_L(**2**)^−^·(CH_3_)_2_NH_2_^+^·DMF; crystal structure data and parameters of the intermolecular interactions for complex H_4_L(**3**) with YbCl_3_ [65], UV-absorption spectroscopy; MALDI-TOF mass spectrometry; luminescence spectroscopy and determination of the *T*1 state energy of the ligand H_4_L(**3**) in its Gd^3+^ complex [66]; computational methodology [67,68,69,70,71,72,73,74,75,76,77,78,79,80]; optimized coordinates of atoms for ligand H_4_L(**3**) and its Yb^3+^ complexes.

## Sample Availability

Samples of all obtained compounds are available from the authors.
