# Supplementary material for: Modulating the Inclusive and Coordinating Ability of Thiacalix[4]arene and Its Antenna Effect on Yb3+-Luminescence via Upper-Rim Substitution
Source: Molecules. 2022 Oct 11;27(20):6793. doi: 10.3390/molecules27206793 (PMC9609577; doi:10.3390/molecules27206793)
Supplement: Supplementary file 1 [file molecules-27-06793-s001.zip › molecules-1908848-supplementary.pdf]

Electronic Supplementary Information

Modulating the inclusive and coordinating ability of thiacalix[4]arenes and their antenna effect on Yb^{3+}-luminescence *via* upper-rim substitution

Author(s): Sergey N. Podyachev,*[a] Svetlana N. Sudakova,[a] Rustem R. Zairov[a], Victor V. Syakaev,[a] Alexey N. Masliy,[b] Michal Dusek,[c] A.T. Gubaidullin,[a] Alexey P. Dovzhenko,[d] Daina N. Buzyurova,[a] Dmitry V. Lapaev,[e] Gulnaz Sh. Mambetova,[a] Vasily M. Babaev,[a] Andrey M. Kuznetsov[b] and Asiya R. Mustafina[a]

[a]*Arbuzov Institute of Organic and Physical Chemistry, FRC Kazan Scientific Center of RAS, Arbuzov str., 8, 420088, Kazan, Russian Federation*
[b] *Kazan National Research Technological University, K. Marx Str., 68, 420015, Kazan, Russian Federation*
[c]*Institute of Physics of the Czech Academy of Sciences, Na Slovance 2, 182 21 Prague, Czech Republic*
[d]*Kazan (Volga region) Federal University, Kremlyovskaya str., 18, 420008 Kazan, Russian Federation*
[e]*Zavoisky Physical-Technical Institute, FRC Kazan Scientific Center of RAS, Sibirsky tract, 10/7, 420029, Kazan, Russian Federation*

Content

1. Structural formulae of the investigated compounds H_4L(**1-3**) S2
2. NMR spectroscopy S2
3. Crystal structure data S3
4. The crystal structure data and the parameters of the intra- and intermolecular interactions for $H_3L(\mathbf{2})^{-}\cdot(CH_3)_2NH_2^{+}\cdot$DMF S4
5. Crystal structure data and parameters of the intermolecular interactions for complex H_4L(**3**) with $YbCl_3$ S7
6. UV-absorption spectroscopy S11
7. MALDI-TOF mass spectrometry S11
8. Luminescence spectroscopy and determination of the T_1 state energy of the ligand H_4L(3) in its Gd^{3+} complex S12
9. Computational methodology S13
10. Optimized coordinates of atoms for ligand H_4L(**3**) and its Yb^{3+} complexes S14

References S27

1. Structural formulae of the investigated compounds H_4L(**1-3**)

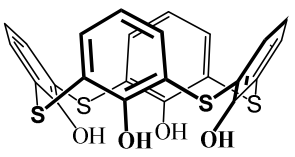

H_4L(**1**)

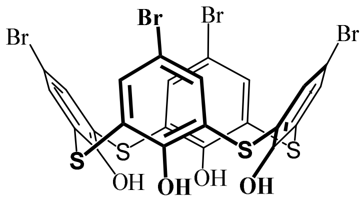

H_4L(**2**)

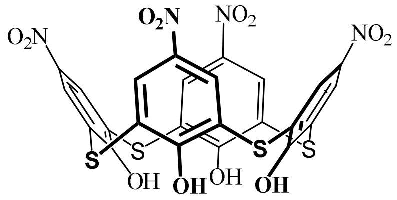

H_4L(**3**)

2. NMR spectroscopy

All NMR experiments were performed on a Bruker AVANCE-500 spectrometer. The spectrometer was equipped with a z-gradient inverse probe head capable of producing gradients with a strength of 50 G cm^{-1}. All experiments were carried out at 303±0.2 K. Chemical shifts (δ) were reported relative to ($CDCl_3$ $\delta(^1H)$ 7.27 ppm, $\delta(^{13}C)$ 77.2 ppm; DMSO $\delta(^1H)$ = 2.50 ppm, $\delta(^{13}C)$ = 39.5 ppm) as an internal standard. The Fourier transform pulsed-gradient spin-echo (FT-PGSE) experiments were performed by BPP-STE-LED (bipolar pulse pair–stimulated echo-longitudinal eddy current delay) sequence [1]. After Fourier transformation and baseline correction, the diffusion dimension was processed with the Bruker TopSpin software package (version 3.2). The diffusion constants were calculated by the exponential fitting of the data belonging to individual columns of the pseudo-2D matrix. Single components have been assumed for the fitting routine. All separated peaks were analyzed, and the average values were presented.

Hydrodynamic radii (r_H) have been calculated from the self-diffusion coefficients D_s applying Stokes –Einstein equation: $D_s = kT/6\pi\eta r_H$, where k -Boltzmann constant (1.38×10^{-23} J/K), T- absolute temperature (303K), η (DMSO, 303K) = 1.73×10^{-3} Pa·s - viscosity of the solvent.

3. Crystal structure data

The X-ray diffraction data for the crystal of compound $H_3L(\mathbf{2})^-\cdot(CH_3)_2NH_2^+\cdot$DMF were collected on a Bruker D8 Quest single crystal X-ray diffractometer equipped with an Incoatec IµS microfocus source (Mo Kα, λ = 0.71073 Å), a multilayers optics monochromator, and a PHOTON III area detector, in the ω and φ-scan modes at 110 K. Data were corrected for absorption effects using the Multi-Scan method by SADABS program [2].

The structures were solved by direct method using SHELXS and refined by the full matrix least-squares using SHELXTL programs.[3] All non-hydrogen atoms were refined anisotropically. The positions of hydrogen atom of OH and NH-groups were found from the electron density-difference map; these hydrogen atoms were refined with individual isotropic displacement parameters. All other hydrogen atoms were placed in ideal calculated positions and refined as riding atoms with relative isotropic displacement parameters. Data collection: images were indexed and integrated using the APEX3 data reduction package [4]. All calculations were performed on PC using WinGX suit of programs [5]. The Mercury program suite was used for molecular graphics.[6] The crystal data, data collection, and the refinement parameters are given in Table S1.

The single crystal of the complex **3** with $YbCl_3$ was measured at the temperature of 95 K on a four-circle diffractometer SuperNova, using a sealed microfocus x-ray tube with a copper anode, a mirror collimator, and CCD detector Atlas S2. We used the program CrysAlis [7] for data collection and reduction, SHELXT [8] and Superflip [9] for structure determination, Jana2020 (the not yet published successor of Jana2006 [10]) for structure refinement and interpretation, and Diamond4 [11] for structure plots.

Crystallographic data (excluding structure factors) for the structures of compound $H_3L(\mathbf{2})^-\cdot(CH_3)_2NH_2^+\cdot$DMF and Yb^{3+} complex with **3** reported in this paper have been deposited in the Cambridge Crystallographic Data Centre as supplementary publication numbers no. CCDC 2177580 and 2166394 respectively. Copies of the data can be obtained free of charge upon application to the CCDC (12 Union Road, Cambridge CB2 1EZ U.K. Fax: (internat.) +44-1223/336-033; E-mail: deposit @ccdc.cam.ac.uk).

4. The crystal structure data and the parameters of the intra- and intermolecular interactions for $H_3L(2)^{-}\cdot(CH_3)_2NH_2^{+}\cdot$DMF

Table S1. Experimental crystallographic data for compound $H_3L(\mathbf{2})^{-}\cdot(CH_3)_2NH_2^{+}\cdot$DMF

Compound<br>Formula	$H_3L(2)^{-}\cdot(CH_3)_2NH_2^{+}\cdot$DMF<br>$C_{24}H_{11}Br_4O_4S_4^{-}$, C_3H_7NO, $C_2H_8N^{+}$
Crystal class	*Triclinic*
Space group	*P-1*
Z, Z′	2, 1
Cell parameters	a = 11.2199(4) Å<br>b = 11.3905(4) Å<br>c = 14.6065(5) Å<br>α = 95.506(1) °<br>β = 103.541(1) °<br>γ = 114.718(1) °
V, $Å^3$	1608.34(10)
M (g/mol)	927.37
T, K	110(2)
Colorless prism-like crystal, Size, mm	0.067x0.129x0.444
F(000)	910
ρ_{calc} g/cm^3	1.915
μ, mm^{-1}	5.307
θ, deg	$1.47 \leq \theta \leq 33.67$
Index ranges	$-17 \leq h \leq 17$,<br>$-17 \leq k \leq 16$,<br>$-22 \leq l \leq 21$
Refl. meas.	50246
Independ/Rint	11143 / 0.0543
Completeness, full	98.9%
Param./restr	413/0
Refl. $[I > 2\sigma(I)]$	9137
R_1/wR_2	0.0444/0.1715
R_1/wR_2 (all refl.)	0.0579/0.1795
Goodness-of-fit	1.308
ρ_{max}/ρ_{min} ($eÅ^{-3}$)	0.839/ -2.402

Table S2. Parameters of H-bonds for $H_3L(\mathbf{2})^{-}\cdot(CH_3)_2NH_2^{+}\cdot$DMF in crystal.

D–H···A	D–H, Å	H···A, Å	D···A, Å	∠ DHA, °	Symmetry operation
O14-H14...S8	0.84	2.57	3.028(3)	115	Intra
O14-H14...O7	0.84	1.73	2.526(3)	157	Intra
O21-H21...S15	0.84	2.60	3.013(2)	112	Intra
O21-H21...O14	0.84	1.77	2.559(4)	155	Intra
O28-H28...S1	0.84	2.73	3.005(2)	101	Intra
O28-H28...O7	0.84	1.83	2.669(4)	176	Intra
N1-H1A...S22	0.91	2.85	3.577(3)	138	
N1-H1A...O21	0.91	2.01	2.756(3)	138	
N1-H1A...O28	0.91	2.23	2.835(3)	123	
N1-H1B...O31	0.91	1.86	2.740(4)	163	

Table S3. Parameters of Br…π contacts for $H_3L(\mathbf{2})^{-}\cdot(CH_3)_2NH_2^{+}\cdot$DMF in crystal.

C-Br···Cg	Br..Cg, Å	C..Cg, Å	∠ Y-X..Cg, °	Symmetry operation
C25-Br4- > Cg1(C2-C7)	3.748(1)	4.712(4)	108.9(1)	1-X,1-Y,1-Z
C25-Br4- > Cg2(C9-C14)	3.822(1)	5.439(3)	142.1(1)	1-X,1-Y,1-Z
C25-Br4- > Cg3(C16-C21)	3.798(1)	4.609(3)	103.1(1)	1-X,1-Y,1-Z
C25-Br4- > Cg4(C23-C28)	3.847(1)	3.941(4)	78.8(1)	1-X,1-Y,1-Z
C18-Br3- > Cg1	3.479(1)	5.350(3)	169.9(1)	1+X,1+Y,Z

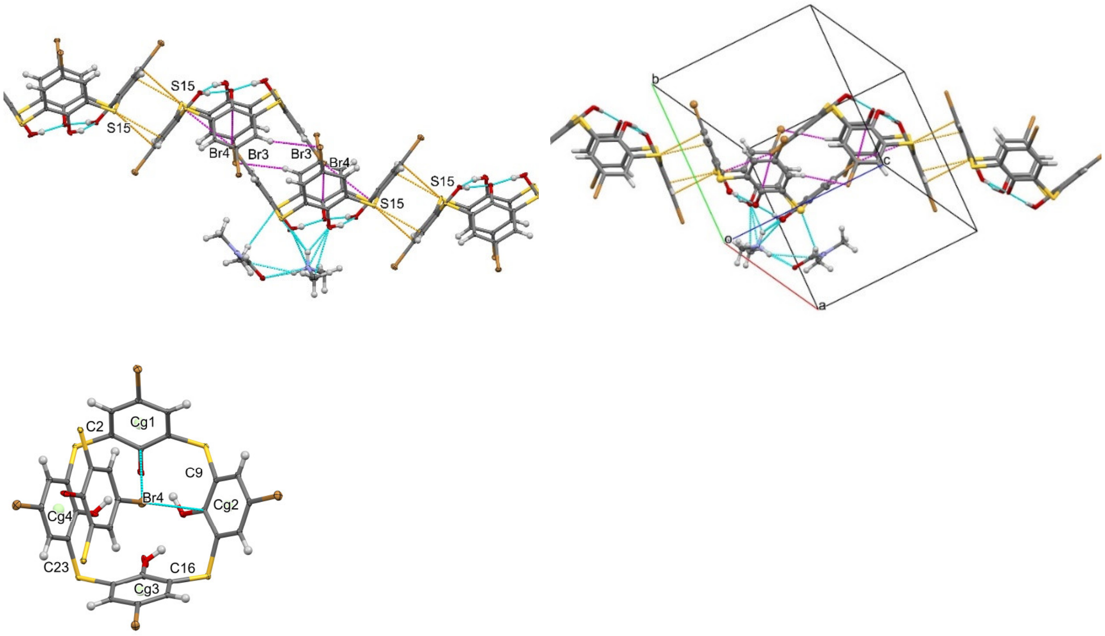

Figure S1. O-H…O, N-H…O and N-H…S hydrogen bonds (blue dashed lines), Br…π and C-H…Br interactions (magenta dashed lines) and π… π and S…π interactions (yellow dashed lines) in the crystal of compound $H_3L(\mathbf{2})^{-}\cdot(CH_3)_2NH_2^{+}\cdot$DMF . Cg1, Cg2, Cg3 and Cg4 - symbols of the centers of aromatic rings C2-C7, C9-C14, C16-C21 and C23-C28, respectively.

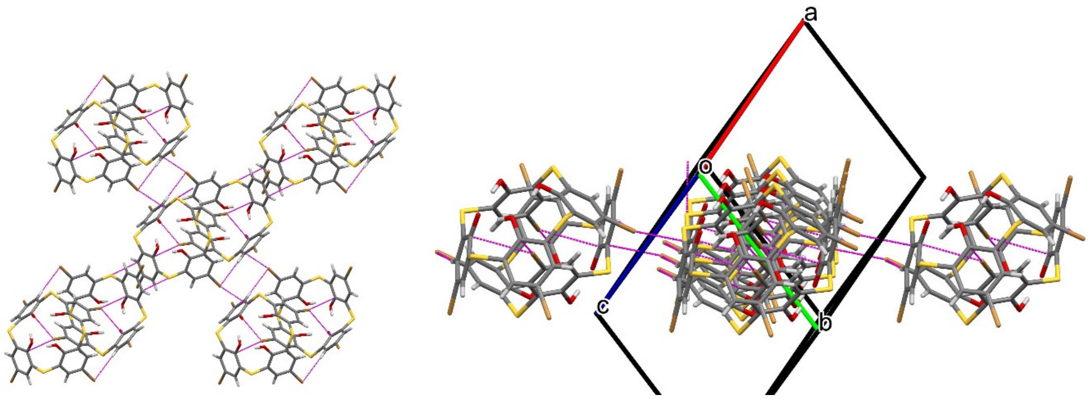

a b

Figure S2. View along (a) [011] and (b) [101] directions on the supramolecular 2D-motives in the crystal of compound $H_3L(\mathbf{2})^{-}\cdot(CH_3)_2NH_2^{+}\cdot$DMF, only Br…π and C-H…Br interactions (magenta dashed lines) are shown.

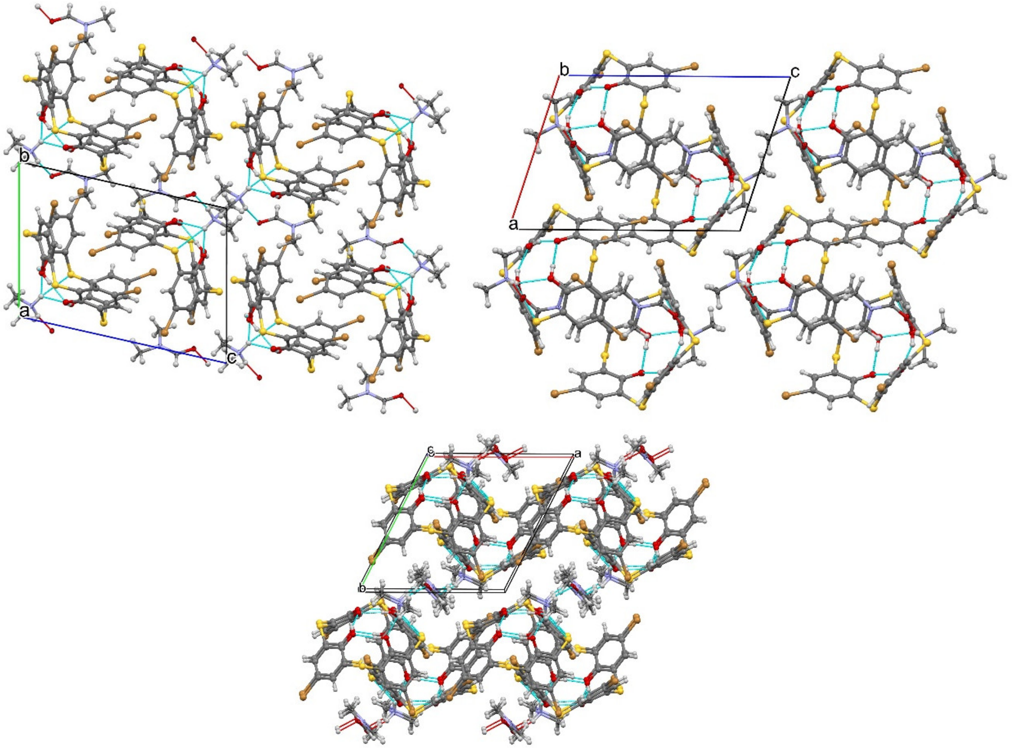

Figure S3. Three projections along main axis of the crystal packing in the crystal of compound $H_3L(\mathbf{2})^{-}\cdot(CH_3)_2NH_2^{+}\cdot$DMF, O-H…O, N-H…O and N-H…S hydrogen bonds are represented by blue dashed lines.

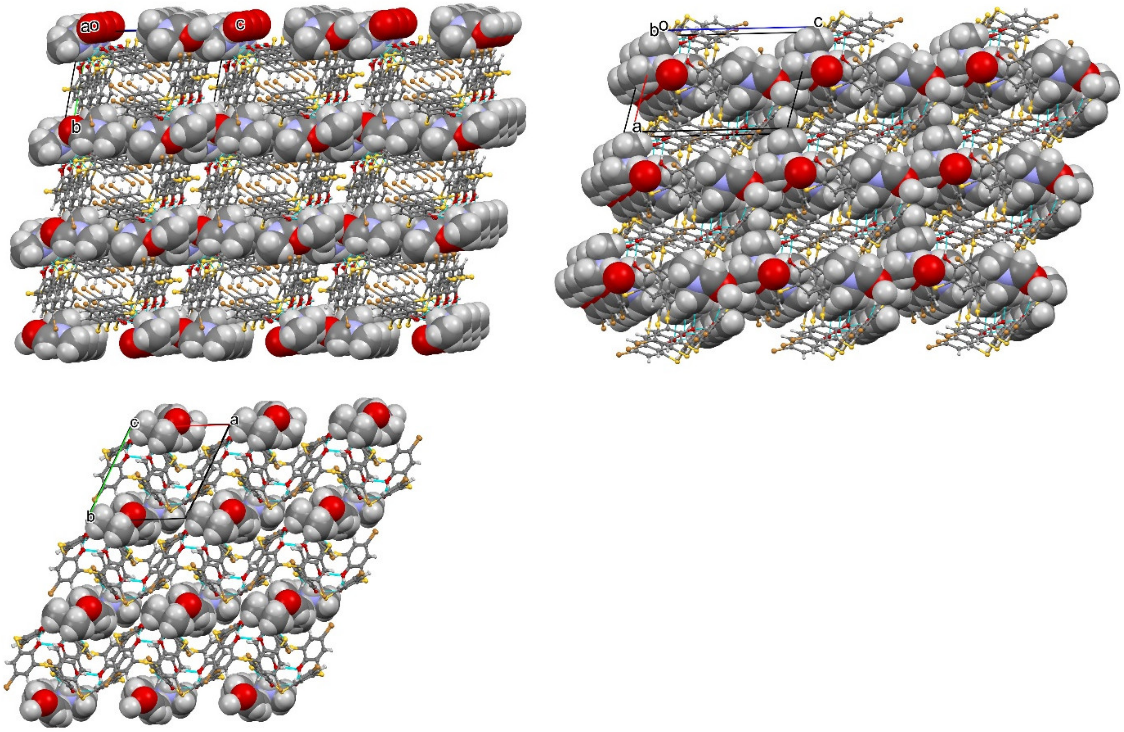

Figure S4. Three projections along main axis of the crystal packing in the crystal of compound $H_3L(\mathbf{2})^{-}\cdot(CH_3)_2NH_2^{+}\cdot$DMF. O-H…O, N-H…O and N-H…S hydrogen bonds are represented by blue dashed lines.

5. Crystal structure data and parameters of the intermolecular interactions for complex $H_4L(\mathbf{3})$ with $YbCl_3$

At the initial attempts to solve the structure, the symmetry was determined as Pn with a pronounced non-centrosymmetry. The molecule's shape was quickly determined, but troubles were encountered with atomic displacement parameters (ADP), especially with the central part of the complex containing chlorine and ytterbium. A closer look at data revealed that the symmetry glide plane *n* is violated, giving rise to many observed reflections with h+l = 2n with intensity between 10 and 20 sigma. Inspection of the CCD frames showed that these reflections were clearly present and could not be explained by a differently oriented piece of the sample, ice peaks or anything else. Therefore, for further calculation, symmetry was lowered to P1, and the structure was refined as a four-fold twin. The twin volume fractions were fixed as ¼ as refinement confirmed they are close to these values. The refined twin fractions close to ¼ correspond with the fact that the merging R-value was good for the monoclinic symmetry, which is thus simulated by the twinning.

Lowering the symmetry to P1 led to two symmetry independent complex molecules. The reason for breaking the glide plane was immediately revealed as the central "cube" made by chlorine and ytterbium was different for both molecules. In one case (see Fig. S5a), Yb and Cl atoms were fully occupied, sulphur and oxygen atoms of ligands bonded to Yb, and slightly disordered Cl atoms bonded only to Yb. In the second case (see Fig. S5b), Yb and Cl atoms were located in mixed sites with Yb:Cl ratio 0.741:0.259 in sited labelled Yb, and opposite ratio in sites labelled Cl. In this second case, due to the presence of Yb in all corners of the "cube", all sites are bonded to oxygen and sulphur atoms of ligands.

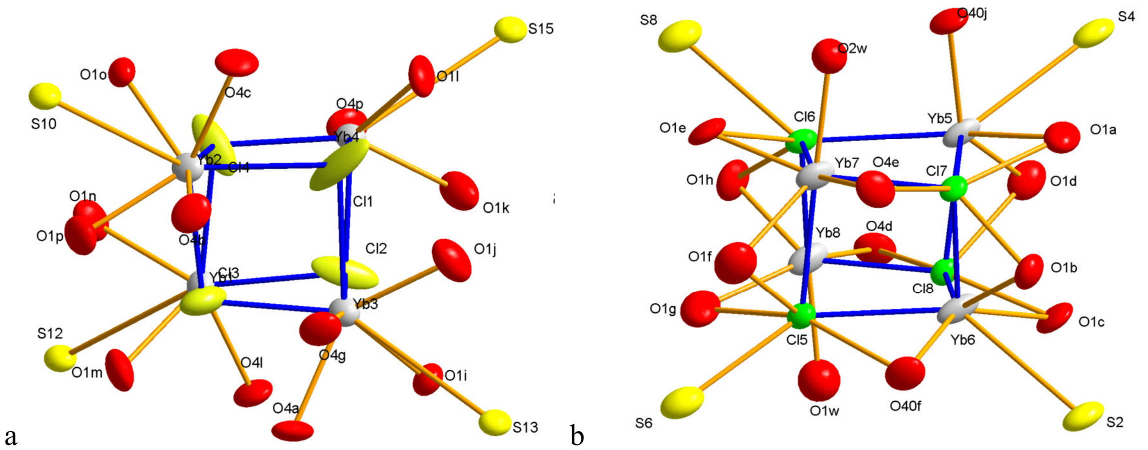

Figure S5. Detailed structure of cubane-like core of the complexes.

Refinement of the triclinic structure model was complicated by disorder and proximity to the Pn symmetry. The refinement program Jana2020 allows connecting atoms by a local symmetry not included in the used space group. Thus, we could connect some atoms by the removed glide plane to decrease correlations of structure parameters. However, this approach would be applicable if the heavy atoms keep the glide plane and the ligands do not. In the present case, the heavy atoms violated the glide plane, and the decision of which light atoms are still keeping the glide plane would be difficult. Therefore, the concept of local symmetry could not be used. Instead, we kept parts of the structure as rigid bodies to stabilize refinement.

The structure model was composed of the following components:

- Rigid body of *p*-nitro-phenolate moiety (Fig. S6a) in sixteen positions, four positions for each of calixarene ring.
- Sixteen individual sulphur atoms, four for each of calixarene ring (Fig. S6b). The ADP of sulphur atoms within one calixarene ring were restricted to be the same.
- Eight individual Yb and Cl atoms to form the central "cubes" – see Fig. S5

- Rigid body of DMF molecule (Fig. S7c) in 19 positions modelling bonded as well as free molecules of DMF
- Two individual oxygen atoms O1w and O2w, representing water coordinated to Yb7 and Yb8

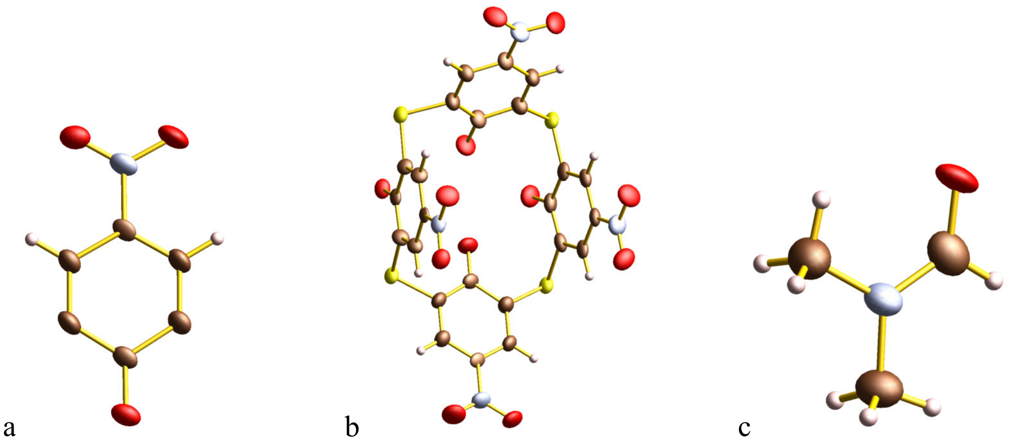

a b c

Figure S6. *a)* *p*-nitro-phenolate moiety, b) *p*-nitro-thiacalix[4]arene anions (L^{4-}) and c) DMF.

These parts altogether formed the structure model, which fitted the experimental data with Robs = 0.0869 and S = 3.30 (please see the note in Table S1 for the meaning of S in the Jana2020 program).

Table S4. Experimental crystallographic data for complex H_4L(**3**) with $YbCl_3$

**Crystal data**	
Chemical formula	$Yb_8S_{16}Cl_8C_{147}O_{67}N_{33}H_{151}$
M_r	5632.89
Crystal system, space group	Triclinic, *P*1
Temperature (K)	95
a, *b*, *c* (Å)	14.682 (3), 17.262 (3), 22.654 (5)
α, β, γ (°)	90.00 (3), 90.00 (3), 105.46 (3)
V (Å^3)	5534 (2)
Z	1
Radiation type	Cu *K*α
μ (mm^{-1})	8.98
Crystal size (mm)	0.21 × 0.07 × 0.05
**Data collection**	
Diffractometer	SuperNova, Dual, Cu at home/near, AtlasS2
Absorption correction	Numerical absorption correction based on gaussian integration over a multifaceted crystal model and empirical absorption correction using spherical harmonics
T_{min}, T_{max}	0.398, 0.942
No. of measured, independent and observed [$I > 3\sigma(I)$] reflections	102370, 42160, 38492
R_{int}	0.036
(sin θ/λ)$_{max}$ (Å^{-1})	0.626
**Refinement**	
$R[F^2 > 3\sigma(F^2)]$, $wR(F^2)$, $S^{1)}$	0.087, 0.231, 3.30
No. of reflections	42160
No. of parameters	595
H-atom treatment	H-atom parameters constrained
$\Delta\rangle_{max}$, $\Delta\rangle_{min}$ (e Å^{-3})	2.70, -1.28

1) Jana software does not refine the weighting scheme to obtain S close to 1. Instead, S is based on the experimental sigma of measured intensities. The value above 3 is expected for a structure where details of disorder are not included in the structure model.

It should be noted that the description of the structure with the above-mentioned rigid bodies is a simplification of the actual situation where many ligand atoms were disordered, structure parameters were correlated due to the almost monoclinic symmetry, and the data resolution was insufficient for modelling structure details. The difference Fourier map indicated several maxima which might belong to free water molecules or their fractions, but refinement did not lead to a convincing decrease of R-factor. In order to detect the disordered positions of rigid bodies, we refined TLS tensors [12], describing rigid-body displacement independently for each position. In cases when the refined displacement was large, we used two close positions of the same rigid body to account for the disorder (see. e.g. positions “s” and “r” of DMF). Finally, TLS tensors were limited to T tensors only because otherwise, the resulting individual ADPs had a non-realistic shape for some atoms.

6. UV-absorption spectroscopy

UV absorption spectra have been recorded on a Lambda 35 spectrophotometer (Perkin-Elmer) in 10 mm quartz cuvettes. The Job plots and spectrometric titrations were done by monitoring absorbance (A_λ). The stoichiometry of the formed complexes Yb^{3+} with H_4L(**1-3**) was studied by means of the isomolar series method (the Job's method) at monitoring the change in the absorbance of the solutions at the wavelength of the complexes' absorption. The obtained results were presented in Figures 5a and 6b as a function A_λ- $A_{\lambda L}\times\alpha_L$ of the α_L, where α_L = $[H_4L]/([H_4L] + [Yb^{3+}]$. A_λ is the absorbance of DMF solution of mixture of $[H_4L]$-TEA–Yb^{3+} ($[Yb^{3+}]$ + $[H_4L]$ = 0.1 mM for H_4L(**1**,**2**) or 0.05 mM for H_4L(**3**), $[H_4L]$:TEA (1:8). $A_{\lambda L}$ is the absorbance of alkalized DMF solution of $[H_4L]$, where $[H_4L]$ = 0.1 mM (0.05 mM) and [TEA] = 0.8 mM (0.04 mM) for H_4L(**1**,**2**) (H_4L(**3**)).

*UV spectral data for complex ligand H_4L(**3**) with $YbCl_3$*

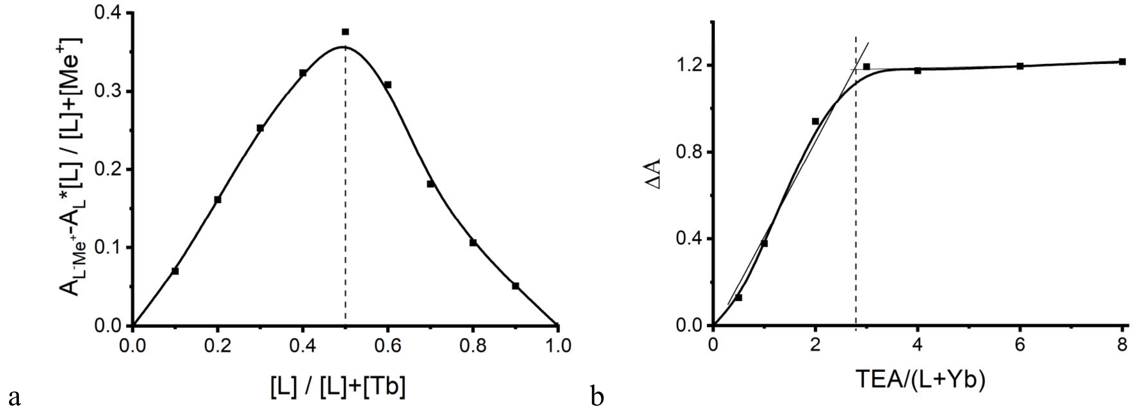

Figure S7. The Job's plot profiles of DMF solutions at the varied H_4L(**3**):$YbCl_3$ molar ratios: (λ =440 nm) ($[Yb^{3+}]$ + [**3**] = 0.05 mM, L:TEA (1:8)) (a) and ΔA of the DMF solutions of H_4L with $YbCl_3$ at the varied TEA:L molar ratio (b) (λ =400 nm, C_{Yb3+} = C_L = 0.05 mM).

7. MALDI-TOF mass spectrometry

The MALDI-TOF mass spectra were obtained on a mass spectrometer UltraFlex III TOF/TOF (Bruker Daltonik GmbH, Germany) in a linear mode, using the laser Nd:YAG, λ = 355 nm. The sample solutions were applied to the metal target MTP AnchorChip™ by the dried drop method. *p*-Nitroaniline (CH_3CN, 10 mg/mL) was used as a matrix. Positively charged ions were registered. The data were obtained using the *FlexControl* program and processed using the *FlexAnalysis 3.0* program.

0.5 μl of the matrix solution and 0.5 μl of the sample were successively applied to the target and evaporated. The samples with molar ratios of 1:1:8, 1:1:10 (L:Yb^{3+}:TEA) were prepared in DMF by the mixing together of corresponding aliquots of ligand (1 × 10^{-4} M), ytterbium nitrate (1 × 10^{-4} M) and TEA (10 × 10^{-4} M).

8. Luminescence spectroscopy and determination of the T_1 state energy of the ligand H_4L(3) in its Gd^{3+} complex

Photoluminescence excitation, emission and time-resolved spectra in NIR range were recorded by an Edinburgh Instruments FLS980 Photoluminescence Spectrometer. A continuous-wave xenon lamp or a microsecond-pulsed xenon flashlamp were used as excitation sources for steady-state or time-resolved measurements, respectively. The excitation wavelength was selected by a double-grating monochromator. The light emitted from the sample was also collected by a double-grating monochromator and recorded by a photon counting R5509-73 PMT cooled at −80 °C (NIR range). Time-resolved PL decays were recorded in multi-channel-scaling (MCS) mode. The measurements were performed at room temperature in aerated conditions.

The time-resolved luminescence spectra of the Gd^{3+} complexes were recorded using an optical spectrometer based on an MDR-23 grating monochromator (LOMO, Saint Petersburg, Russia) coupled to a FEU-100 photomultiplier tube.[13] The luminescence was excited by an LGI-21 pulsed nitrogen laser (337 nm wavelength, 2.1 mW laser pulse average output power, 10 ns pulse duration, 100 Hz repetition rate). The average output power of the laser near the samples was 1.7 mW. The exposed surface areas of the samples were 7 mm^2. The measurements were performed at different temperatures in aerated conditions.

The phosphorescence spectra of the Gd^{3+} complexes with H_4L(**3**) at 143 K and 40 μs time delay are characterized by broad bands with maximum at 589 nm (16978 cm^{-1}) (Figure S8). Since these spectra do not contain a fine vibrational structure, required to accurate determination of the position of the maximum of the 0–0 phonon transition corresponding to the energy of the T_1 of the state of the ligands, we used a spectral deconvolution of the spectra into a series of overlapping bands using a Gaussian function. Energies values of the T_1 states of the ligands were determined from the maxima of short-wavelength deconvoluted bands. The value for the Gd^{3+} complex with **3** was 567 nm (17637 cm^{-1}).

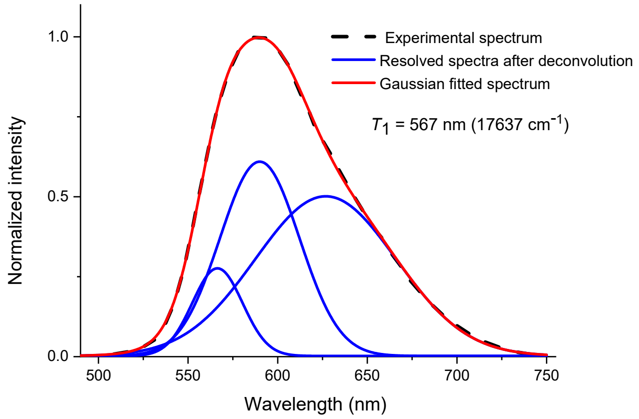

Figure S8. A spectral deconvolution of the normalized phosphorescence spectra of the Gd^{3+} complexes with **3** at 40 μs time delay and T = 143 K into subcomponent by using a Gaussian curve fitting. $C_L = C_{Gd3+}$ = 1 mM, C_{TEA} = 4 mM.

9. Computational methodology

Quantum-chemical modeling of the Yb^{3+} complex structures with H_4L (**3**) was carried out using the PRIRODA program package [14] within the density functional theory employing the generalized gradient approximation PBE functional [15] For all atoms, a specially optimized double-zeta atomic basis set L1 (similar to cc-pVDZ) by Laikov [16] was applied. The PBE functional has demonstrated its reliability in our early theoretical studies of several supramolecular systems [17–22].

All computations were performed with full gas-phase optimization of molecular geometry without any symmetry constraints. Optimized coordinates of atoms for ligand **3** and their Yb^{3+} complexes are available in Supplementary. The geometry optimization was followed by calculations of vibrational spectra. Normal mode vibrational frequencies were calculated in the harmonic approximation without using any scaling factors. The absence of imaginary values in the frequency spectrum of normal modes was deemed to confirm that the obtained structures correspond to the minimum on the total potential energy surface. Based on thermochemical analysis (at 298 K temperature and 1 Atm pressure), the total entropy, enthalpy and Gibbs free energy of each system were obtained.

Since the PRIRODA program package does not allow the possibility of carrying out quantum-chemical calculations taking into account the influence of a solvent, we used the following approach to evaluate solvent effects. Using a fixed geometry of particles involved in the reaction, previously optimized with the PRIRODA program, their total energy in the gas phase and in solution was calculated using the Gaussian09 [23] program package within the Polarizable Continuum Model (PCM) [24–26]. The solvation energy of a particle was calculated

as the difference between the total energy in the gas phase and in solution, and then it was added to the total gas-phase energy of the particle obtained with the PRIRODA program package. Since the atomic basis set L1 is not implemented in the Gaussian09 basis set library, we used for all the atoms the x2c-SVPAll basis set of a similar size [27].

10. Optimized coordinates of atoms for ligand $H_4L(3)$ and its Yb^{3+} complexes

$[Yb(H_2O)_8]^{3+}$

```
    25
[Yb(H2O)8]3+ complex optimized structure
 O     2.386397    -2.170776    -6.794786
Yb     0.662318    -2.755302    -5.271743
 O     0.188755    -3.024362    -2.953163
 O     2.633822    -3.815781    -4.483485
 O     1.775624    -0.996473    -4.113926
 O    -0.092811    -3.423577    -7.420350
 O    -1.706765    -2.729317    -5.025975
 O    -0.121628    -0.699744    -6.184796
 O     0.155113    -5.068256    -5.109322
 H     0.058379    -5.708526    -5.848992
 H     0.071587    -5.601359    -4.287615
 H    -2.308145    -3.480969    -4.827925
 H    -2.290668    -1.947776    -5.144959
 H     0.788281    -3.336453    -2.239683
 H    -0.682031    -2.878154    -2.521571
 H     1.629391    -0.737765    -3.177126
 H     2.491726    -0.408775    -4.442037
 H     3.430118    -3.381782    -4.104092
 H     2.855928    -4.771946    -4.536975
 H    -0.606063    -0.550993    -7.026862
 H     0.017590     0.193260    -5.798686
 H     2.354326    -1.475066    -7.488576
 H     3.251375    -2.622184    -6.915067
 H    -1.004176    -3.693610    -7.671114
 H     0.453171    -3.560820    -8.226263
```

$H_4L(3)$

```
    56
 H4L ligand  optimized structure
 C    -0.110338     2.772277    -1.333820
 C     1.094036     3.245263    -0.756212
 C     1.048576     4.090670     0.350380
 C    -0.182888     4.458412     0.880970
 C    -1.379106     4.014361     0.326396
 C    -1.351197     3.165700    -0.776806
 S     2.670213     2.894839    -1.560356
 C     3.181835     1.347229    -0.785655
 C     2.777279     0.107026    -1.336304
 C     3.265932    -1.097465    -0.772087
 C     4.140386    -1.052837     0.311758
 C     4.521681     0.178122     0.833766
 C     4.060241     1.374519     0.294083
 S     2.895179    -2.673721    -1.566140
 C     1.348507    -3.165027    -0.776075
```

```
C     1.375292    -4.012290     0.328284
C     0.178541    -4.456344     0.881676
C    -1.052406    -4.090334     0.348694
C    -1.096672    -3.246118    -0.758812
C     0.108180    -2.772756    -1.334915
N     0.217394    -5.361246     2.059975
O     0.115705    -1.956830    -2.407604
S    -2.671476    -2.895014    -1.565231
C    -3.182261    -1.348042    -0.788951
C    -4.055416    -1.376100     0.294997
C    -4.515079    -0.180086     0.837098
C    -4.136697     1.051293     0.313913
H     0.453171    -3.560820    -8.226263
```

Triethylamine (TEA)

```
    22
 TEA  optimized structure
   7     0.10579392   0.18327350   0.07482940
   6     0.22700294   0.06140982   1.53307926
   6     1.42524109   0.22710895  -0.56793248
   6    -0.70746185   1.34788685  -0.29705198
   6     1.38962287  -0.15385587  -2.04970479
   1     1.90777999   1.22665685  -0.44221078
   1     2.07491690  -0.49364787  -0.04597491
   1    -0.53154049   1.55041056  -1.36571954
   6    -2.20851789   1.12625190  -0.09768054
   1    -0.38100748   2.26547159   0.24994578
   1    -0.75138481   0.31506530   1.97175150
   6     0.60124214  -1.34927161   1.99346005
   1     0.95318799   0.80300193   1.94592330
   1     0.63217531  -1.39101099   3.09435107
   1    -0.14115019  -2.07802354   1.63322883
   1     1.59136232  -1.66065597   1.62500473
   1     2.41051776  -0.14902515  -2.46499710
   1     0.96230648  -1.16069864  -2.17492176
   1     0.78826549   0.54864711  -2.64797155
   1    -2.76894366   2.01311261  -0.43540795
   1    -2.54594335   0.25341296  -0.67758460
   1    -2.46945946   0.95540472   0.95862506
```

HTEA$^+$ (protonated form of TEA)

```
    23
HTEA+ optimized structure
   7     0.11021667   0.19012816   0.07725661
   6     0.24198302   0.04041804   1.59006389
   6     1.47082798   0.23047086  -0.61113726
   6    -0.75514135   1.38797515  -0.30211185
   6     1.39632434  -0.18051785  -2.07257915
   1     1.84507082   1.25771736  -0.48034112
   1     2.12528982  -0.45041915  -0.04878323
   1    -0.53130648   1.61360444  -1.35429554
   6    -2.23898272   1.11779555  -0.11408582
   1    -0.39738966   2.22624797   0.31571730
   1    -0.72186898   0.34852619   2.01919996
   6     0.58691061  -1.37991812   2.00661076
   1     1.00777255   0.76857102   1.89940455
   1     0.63759129  -1.41527904   3.10560878
   1    -0.19069961  -2.09472205   1.69183732
   1     1.56140609  -1.71794028   1.62477283
   1     2.41395682  -0.15505429  -2.49133978
```

```
   1     1.02204142  -1.21105140  -2.18505549
   1     0.77698860   0.49495859  -2.68072874
   1    -2.79625704   2.01168945  -0.43348448
   1    -2.58066708   0.27805696  -0.74081909
   1    -2.51333890   0.91987528   0.93245162
   1    -0.37812691  -0.65670296  -0.26845774
```

DMF

```
    12
DMF optimized structure
   N     0.27471019  -0.40389703   0.14127218
   C     0.10690714  -0.14265072   1.55573429
   C     1.28409048   0.17411430  -0.58639911
   C    -0.66131721  -1.30546494  -0.50714125
   H    -0.36784212  -1.37118332  -1.56484982
   H    -0.62470557  -2.30978610  -0.04953199
   H    -1.69470198  -0.92341690  -0.43255487
   H    -0.87722753   0.31342894   1.76673675
   H     0.19130178  -1.07094825   2.14919333
   H     0.89094860   0.55491214   1.89018937
   O     1.49333600   0.00736780  -1.77452039
   H     1.90726222   0.83553307   0.06676450
```

[YbHL(DMF)$_4$]-(I)

```
    102
[YbHL(DMF)4]-(I) complex optimized structure
 C    -0.194388     0.195608     2.582066
 C    -1.493112     0.751037     2.839539
 C    -1.658589     2.043490     3.320266
 C    -0.536752     2.828610     3.578550
 C     0.752220     2.328031     3.388243
 C     0.922548     1.041688     2.898390
 S    -2.932541    -0.307458     2.654082
 C    -3.675364     0.249054     1.125598
 C    -2.964492     0.175687    -0.135350
 C    -3.690864     0.723540    -1.264777
 C    -5.011420     1.139793    -1.169329
 C    -5.673806     1.074349     0.056044
 C    -4.999106     0.668243     1.203894
 S    -2.947581     0.767548    -2.893627
 C    -1.562367     1.880742    -2.622775
 C    -1.790013     3.235704    -2.394187
 C    -0.712860     4.101952    -2.231278
 C     0.603437     3.657182    -2.317568
 C     0.837922     2.306725    -2.545883
 C    -0.238295     1.398471    -2.691798
 N    -0.973354     5.544309    -2.008343
 O    -0.015268     0.084736    -2.880923
Yb     0.137208    -1.438678    -0.085499
 O    -0.969615    -3.431329     0.728316
 S     2.490402     1.644277    -2.758360
 C     3.184775     1.662930    -1.104593
 C     4.457400     2.211126    -0.997609
 C     5.127274     2.165701     0.223473
 C     4.506844     1.645145     1.358384
 C     3.231794     1.098700     1.268575
 C     2.516513     1.023214     0.009590
 O    -1.791701    -0.335007    -0.258147
 N    -7.079351     1.486643     0.144901
```

N	6.486378	2.710252	0.322056
S	2.581790	0.365986	2.762549
O	1.384693	0.430632	-0.116638
N	-0.712080	4.191107	4.108568
O	-0.042139	-0.994075	2.094762
O	1.864932	-2.856595	0.839823
O	1.960744	-1.777082	-1.812266
O	-0.634843	-2.765741	-1.899743
H	-2.804392	3.633460	-2.343494
H	1.419405	4.372559	-2.209870
H	-5.524409	0.696722	2.159923
H	1.604969	2.963618	3.631751
H	-2.649231	2.456635	3.516626
H	4.949247	2.682228	-1.850319
H	5.035320	1.689095	2.312264
H	-5.547784	1.525266	-2.037645
H	0.974137	-0.047731	-2.818601
O	-7.630284	1.859998	-0.899327
O	-7.622521	1.425001	1.255592
O	0.311587	4.846585	4.337323
O	-1.869756	4.584499	4.292772
O	0.009843	6.280737	-1.891603
O	-2.152726	5.902394	-1.961375
O	7.036834	2.673951	1.430245
O	6.998362	3.156053	-0.714438
C	3.185899	-1.739137	-1.618005
C	2.075811	-2.987454	2.060376
C	-1.807380	-3.522748	1.642248
C	-1.506902	-2.575455	-2.764735
H	3.619787	-1.682826	-0.600729
H	1.480480	-2.419975	2.797703
H	-2.148996	-2.631621	2.206678
H	-2.057045	-1.617372	-2.843517
N	-1.861230	-3.492624	-3.687722
N	4.121230	-1.761053	-2.588639
N	3.013229	-3.800295	2.583861
N	-2.380841	-4.681987	2.027057
C	-2.051033	-5.941639	1.375523
C	-3.375583	-4.706043	3.089699
C	-1.227413	-4.802557	-3.733236
C	-2.882106	-3.194962	-4.681996
C	3.747264	-1.844058	-3.994688
C	5.534882	-1.606614	-2.275791
C	3.878903	-4.604666	1.732283
C	3.246862	-3.844204	4.020136
H	3.121743	-4.871287	4.402474
H	4.267508	-3.500300	4.258929
H	2.528436	-3.184162	4.526513
H	3.840880	-5.661540	2.043744
H	3.521073	-4.509851	0.698479
H	4.923802	-4.254840	1.798192
H	-4.340528	-5.083112	2.710224
H	-3.045465	-5.356616	3.917400
H	-3.521802	-3.686837	3.474655
H	-2.946331	-6.373693	0.896567
H	-1.286636	-5.736752	0.613971
H	-1.661642	-6.664989	2.111909
H	-2.465910	-3.275048	-5.700679
H	-3.729175	-3.896239	-4.592494
H	-3.249741	-2.170234	-4.530303
H	-0.713554	-4.948601	-4.698819
H	-0.497482	-4.852221	-2.914283
H	-1.981971	-5.597961	-3.611326

```
 H     6.113077    -2.466144    -2.654362
 H     5.932557    -0.683659    -2.730182
 H     5.663533    -1.537978    -1.186087
 H     4.287637    -2.674620    -4.478240
 H     2.665340    -2.022616    -4.051147
 H     3.991493    -0.904548    -4.518068
```

[YbHL(DMF)$_5$]-(II)

```
    114
[YbHL(DMF)5]-(II) complex optimized structure
  C    -1.815753     4.259832    -1.395838
 C    -1.311462     2.982967    -1.601093
 C    -1.256459     2.020176    -0.517555
 C    -1.565562     2.537284     0.793299
 C    -2.081943     3.809321     0.981475
 C    -2.251980     4.648911    -0.123045
 S    -0.851340     2.459168    -3.250423
 C     0.898461     2.145138    -2.987799
 C     1.691276     3.171888    -2.507046
 C     3.046049     2.945850    -2.256869
 C     3.625782     1.693858    -2.477676
 C     2.848993     0.654945    -2.953093
 C     1.427088     0.809520    -3.270274
 S     3.583221    -0.965116    -3.188442
 C     4.338467    -1.207463    -1.581412
 C     3.509699    -1.267569    -0.418526
 C     4.130418    -1.469103     0.843145
 C     5.507975    -1.664184     0.927369
 C     6.278192    -1.662497    -0.232434
 C     5.709450    -1.415904    -1.481945
 O     2.205984    -1.137911    -0.592271
 S     3.165473    -1.668603     2.349536
 C     2.324364    -0.095927     2.614770
 C     0.988921     0.081637     2.102706
 C     0.281113     1.239385     2.570247
 C     0.876783     2.168263     3.412857
 C     2.193470     1.980336     3.828068
 C     2.911986     0.846758     3.449175
 N     7.729885    -1.899510    -0.133010
 O     8.197885    -2.095936     0.994462
 O     0.473036    -0.779279     1.276880
Yb    -1.685988    -1.039700     0.399975
 O    -0.457402    -2.648724    -0.728933
 S    -1.471330     1.403274     2.188331
 N     2.812595     2.963808     4.732518
 O     3.972741     2.745558     5.099221
 N     3.884037     4.052491    -1.781308
 O     3.337937     5.152170    -1.616542
 O     0.726823    -0.133095    -3.729322
 O    -0.986762     0.784300    -0.707254
 N    -2.917417     5.935943     0.054401
 O    -3.477937     6.140957     1.147161
 O    -1.784311    -1.925252     2.640445
 O    -3.875047    -0.114429     0.747779
 O    -3.113375    -3.040528     0.428950
 O    -2.819478    -1.149431    -1.791042
 O     2.125313     3.935064     5.070118
 O     8.378389    -1.893844    -1.185511
 O    -2.912131     6.727616    -0.896493
 O     5.082283     3.813562    -1.577044
 H    -2.370672     4.171558     1.969823
 H    -1.899480     4.971969    -2.218947
```

H	3.923585	0.710247	3.834005
H	6.352385	-1.381673	-2.362310
H	5.997247	-1.826520	1.888425
H	1.287729	4.166923	-2.314218
H	4.688186	1.569699	-2.263034
H	0.334666	3.046804	3.766664
H	1.639878	-1.124838	0.263655
C	-4.267291	-3.170919	0.005369
C	0.173992	-2.712270	-1.808478
C	-2.301811	-0.794307	-2.872720
C	-4.499643	0.811649	1.296872
C	-0.856782	-2.448536	3.284701
H	-1.294566	-0.339228	-2.941524
H	0.413477	-1.822682	-2.423010
H	0.155851	-2.571130	2.853829
H	-4.842143	-2.321246	-0.412110
H	-4.619985	0.864277	2.398655
N	-5.093678	1.830908	0.657829
N	-2.900763	-0.915422	-4.074321
N	0.628674	-3.860977	-2.335209
N	-0.966243	-2.901010	4.547716
N	-4.954618	-4.335624	0.000365
C	0.186763	-3.449756	5.248898
C	-2.219418	-2.789570	5.282213
C	-4.351393	-5.561029	0.506225
C	-6.312455	-4.411042	-0.512661
C	-5.001604	1.959348	-0.794010
C	-5.713639	2.930020	1.391641
C	-4.248273	-1.443556	-4.208764
C	-2.210841	-0.475359	-5.283776
C	0.450825	-5.134783	-1.653536
C	1.320164	-3.861460	-3.619463
H	2.323149	-4.305438	-3.511386
H	0.749658	-4.447413	-4.360349
H	1.431649	-2.823611	-3.965539
H	1.428696	-5.620804	-1.503057
H	-0.024671	-4.938268	-0.683471
H	-0.189873	-5.806413	-2.250797
H	-7.007786	-4.758641	0.270503
H	-6.632060	-3.413526	-0.847967
H	-6.371416	-5.107461	-1.366712
H	-4.945075	-5.965244	1.343469
H	-4.298622	-6.321706	-0.291019
H	-3.338223	-5.318180	0.853300
H	-4.253139	-2.323772	-4.874267
H	-4.600618	-1.733869	-3.209730
H	-4.923080	-0.680333	-4.633874
H	-2.158016	-1.305043	-6.009178
H	-2.747862	0.367447	-5.751161
H	-1.191099	-0.158917	-5.014064
H	-0.014018	-4.484149	5.574453
H	1.057642	-3.448444	4.577677
H	0.424858	-2.839355	6.136079
H	-2.533769	-3.780196	5.650220
H	-2.100319	-2.111528	6.144310
H	-2.977497	-2.385574	4.598498
H	-6.752459	3.074445	1.053564
H	-5.718448	2.695712	2.465972
H	-5.148956	3.865360	1.236828
H	-6.008486	2.106512	-1.216553
H	-4.366191	2.820886	-1.057622
H	-4.549719	1.041081	-1.194135

[Yb$_2$HL$_2$(DMF)$_4$] -(III)

```
   156
[Yb2HL2(DMF)4]-(III) complex optimized structure
 C    -4.937795    -1.507051    -4.361517
 C    -5.086044    -2.006083    -3.064798
 C    -4.112020    -1.736733    -2.114471
 C    -2.913463    -1.004565    -2.443337
 C    -2.901408    -0.375881    -3.747798
 C    -3.874702    -0.663208    -4.694307
 S    -4.247570    -2.477463    -0.477426
 C    -4.713997    -1.138617     0.639407
 C    -3.694415    -0.496847     1.417230
 C    -4.144824     0.359889     2.479010
 C    -5.493759     0.634243     2.667573
 C    -6.438396     0.042971     1.830812
 C    -6.060524    -0.864419     0.842527
 S    -1.592685     0.794976    -4.089155
 C    -2.201008     2.114420    -3.031550
 C    -3.506719     2.571583    -3.176441
 C    -3.986756     3.588668    -2.356895
 C    -3.177730     4.180133    -1.385236
 C    -1.881350     3.722258    -1.232524
 C    -1.320300     2.672705    -2.041691
 O    -1.905538    -0.939770    -1.652569
Yb    -1.336112    -2.293877     0.025300
 O    -1.752892    -4.014591    -1.536958
 S    -2.945066     0.892337     3.711055
 C    -2.418516     2.516965     3.160686
 C    -2.552539     3.568778     4.065228
 C    -2.085353     4.837568     3.731551
 C    -1.587428     5.105827     2.459596
 C    -1.456290     4.066721     1.546138
 C    -1.780706     2.725380     1.904821
 N    -7.871056     0.330093     2.037747
 O    -2.421713    -0.674880     1.209615
 S    -0.765175     4.479679    -0.058010
Yb     1.562933     2.666582    -0.473683
 O     2.111560     4.594376     0.830208
 O    -1.468071     1.767464     1.044291
 N    -5.365912     4.075701    -2.539998
 O    -0.107968     2.262292    -1.886769
 S     0.554707    -4.537876     0.537974
 C     1.040570    -3.846946     2.123086
 C     1.371577    -4.689038     3.171440
 C     1.611596    -4.137652     4.432527
 C     1.617738    -2.752645     4.624970
 C     1.309863    -1.899359     3.573826
 C     0.884603    -2.421836     2.292327
 N     1.860563    -5.030312     5.569633
 S     1.365810    -0.122047     3.788959
 C     2.948676     0.182886     2.994967
 C     4.092528    -0.471599     3.435509
 C     5.317515    -0.218599     2.825028
 C     5.433544     0.695258     1.777401
 C     4.299656     1.347819     1.322930
 C     2.998266     1.119954     1.901701
 S     4.454902     2.541985    -0.028687
 C     4.759234     1.450775    -1.421290
 C     6.007512     1.404122    -2.018948
 C     6.208608     0.555921    -3.110443
 C     5.192152    -0.286813    -3.559456
```

C	3.943289	-0.255313	-2.951333
C	3.637522	0.681996	-1.901792
N	7.513833	0.537770	-3.784876
S	2.668005	-1.382772	-3.531053
C	2.880469	-2.736613	-2.357631
C	4.015999	-3.539891	-2.352581
C	4.096461	-4.591397	-1.446537
C	3.062038	-4.877312	-0.563387
C	1.919595	-4.078328	-0.554008
C	1.825582	-2.990984	-1.449464
O	0.723648	-2.196300	-1.457629
O	2.451297	0.839863	-1.429332
O	1.933211	1.707669	1.467858
O	0.360454	-1.688868	1.381054
O	-2.123515	-3.572348	1.825531
O	2.407037	3.942768	-2.221634
H	4.843566	-3.369836	-3.042593
H	3.161355	-5.730489	0.109482
H	6.840919	2.012908	-1.663463
H	4.062579	-1.185849	4.259599
H	6.418625	0.874680	1.343194
H	1.431570	-5.772200	3.050244
H	1.872100	-2.363928	5.612754
H	5.404800	-0.959033	-4.392512
H	-5.968204	-2.605163	-2.831120
H	-3.842626	-0.229347	-5.695293
H	-5.836174	1.287415	3.471519
H	-1.319921	6.129457	2.194615
H	-3.014187	3.410680	5.040918
H	-4.179656	2.151669	-3.925441
H	-3.588447	4.984808	-0.773201
H	-6.839662	-1.349872	0.252070
H	-1.766478	0.824135	1.312627
H	0.942839	-1.398589	-2.026509
N	-2.144217	5.922413	4.722523
N	-5.939297	-1.846093	-5.382743
N	6.527918	-0.902788	3.320176
N	5.302303	-5.460852	-1.452006
O	5.318444	-6.398788	-0.652683
O	6.189561	-5.179318	-2.259285
O	7.649193	-0.237077	-4.739756
O	8.383257	1.306359	-3.356337
O	7.597648	-0.629941	2.764328
O	6.385882	-1.698893	4.254048
O	2.096510	-4.506759	6.665485
O	1.791879	-6.250070	5.359699
O	-2.742649	5.703328	5.777806
O	-1.565880	6.983157	4.433701
O	-5.745712	4.987293	-1.795937
O	-6.043327	3.541598	-3.424549
O	-8.675036	-0.245982	1.297524
O	-8.163334	1.122077	2.939528
O	-5.783541	-1.366303	-6.512165
O	-6.859048	-2.602917	-5.046266
C	2.195102	3.768988	-3.441984
C	2.031235	4.772216	2.063326
H	1.462711	3.019630	-3.799067
H	1.927740	3.921257	2.764704
N	2.047880	5.971300	2.663211
C	1.877549	6.098359	4.107647
C	2.149628	7.203266	1.888215
N	2.808529	4.451145	-4.416618
C	2.510688	4.192384	-5.820538

C	3.814759	5.465008	-4.113635
C	-1.874594	-4.098942	-2.775252
C	-2.200674	-3.205581	3.020701
H	-1.605167	-3.264274	-3.450883
H	-2.161782	-2.134306	3.298037
N	-2.326878	-4.034670	4.062715
N	-2.333273	-5.179348	-3.422774
C	-2.388383	-3.519775	5.426749
C	-2.358752	-5.483789	3.887777
C	-2.482651	-5.182075	-4.873577
C	-2.752228	-6.374946	-2.698306
H	3.419452	3.861020	-6.348851
H	1.747106	3.405471	-5.895647
H	2.129508	5.106327	-6.304441
H	3.908059	5.542322	-3.023082
H	4.784206	5.174978	-4.550397
H	3.507520	6.436707	-4.533204
H	1.254838	7.826142	2.051688
H	3.041336	7.771933	2.199038
H	2.230272	6.936675	0.826473
H	0.934915	6.624888	4.334200
H	1.845931	5.097479	4.561192
H	2.722391	6.657375	4.541515
H	-1.873227	-5.984554	-5.320312
H	-3.538663	-5.340048	-5.146861
H	-2.154733	-4.214808	-5.279481
H	-2.557628	-6.217773	-1.629822
H	-3.828631	-6.554372	-2.855970
H	-2.186099	-7.249470	-3.057528
H	-2.306288	-5.703391	2.813818
H	-1.501523	-5.943959	4.405805
H	-3.294096	-5.891441	4.304583
H	-1.541791	-3.906441	6.016912
H	-2.337319	-2.422123	5.407859
H	-3.331278	-3.826487	5.907832

[YbHL(OH)(DMF)3]$^{-}$-(IV)

92
[YbHL(OH)(DMF)3]- -(IV) complex optimized structure

C	-0.07947925	-1.52964241	1.81617571
C	-1.20679370	-0.85102824	2.39972472
C	-1.04156442	0.15543441	3.33773886
C	0.24600131	0.54400389	3.72445513
C	1.37652132	-0.08531477	3.19332503
C	1.22085327	-1.09639481	2.25858880
S	-2.85956264	-1.43121498	1.97783638
C	-3.51545599	-0.16147534	0.89549510
C	-2.89886911	0.11206452	-0.38429651
C	-3.57847493	1.08774837	-1.20694629
C	-4.77363351	1.67724282	-0.81766783
C	-5.35162911	1.33307498	0.40555883
C	-4.71314352	0.43547216	1.26788509
S	-2.95812890	1.42900529	-2.85350698
C	-1.32149906	2.08073174	-2.49116286
C	-1.18518830	3.31069587	-1.85551659
C	0.08530932	3.82205533	-1.58905282
C	1.23848042	3.13327358	-1.95613340
C	1.11128430	1.90508497	-2.59165747
C	-0.16500994	1.35888119	-2.86848610
N	0.22051776	5.14429438	-0.95028525
O	-0.29369189	0.17275591	-3.47217787
Yb	-0.50368988	-2.18904548	-1.25343488

O	-2.16395663	-3.45564874	-0.92501360
S	2.52417224	0.96883267	-3.17633658
C	3.30255491	0.35939993	-1.67934888
C	4.66631089	0.59332572	-1.57121690
C	5.38209477	0.04163632	-0.50809166
C	4.71807790	-0.68194550	0.48963936
C	3.35436498	-0.92548549	0.39810539
C	2.55982790	-0.45627029	-0.72994825
O	-1.82733313	-0.46616328	-0.78839056
N	-6.61799071	1.94088722	0.79176523
N	6.81814488	0.25255240	-0.42396116
S	2.65767181	-1.99318435	1.65480232
O	1.32675917	-0.73733682	-0.89165574
N	0.41690091	1.58833042	4.73196710
O	-0.21519190	-2.47253817	0.95359633
O	0.78732639	-4.27951685	-1.16756933
O	1.16719232	-2.33987594	-3.25744804
O	-1.61430331	-2.38957460	-3.41010869
H	-2.06145755	3.88698233	-1.55726362
H	2.21508219	3.56962098	-1.74366511
H	-5.17344048	0.21629118	2.23192910
H	2.36486719	0.22941577	3.53144395
H	-1.90097484	0.65241410	3.78925533
H	5.19928932	1.20092654	-2.30431407
H	5.29599414	-1.04558049	1.34089501
H	-5.27810934	2.40741934	-1.45235048
H	0.61500569	-0.22662033	-3.55665807
O	-7.13985682	2.74133183	-0.00263019
O	-7.10774687	1.61929919	1.88698495
O	1.57659969	1.88332328	5.06199589
O	-0.60443366	2.11143000	5.20353206
O	1.36823600	5.56297055	-0.75794974
O	-0.81677845	5.75167026	-0.66018120
O	7.42425217	-0.24388858	0.54139381
O	7.36171460	0.90424002	-1.33404430
C	2.36126238	-2.62163580	-3.09041793
C	0.93151430	-4.88066356	-0.09007031
H	-2.44144813	-3.30234348	-0.00148668
C	-2.84386038	-2.22337522	-3.36420191
H	2.76417951	-2.97950080	-2.12235550
H	0.70656645	-4.38464962	0.87320318
H	-3.36415801	-1.96543649	-2.42411166
N	-3.65650691	-2.34491567	-4.43781884
N	3.31399638	-2.53406632	-4.04920979
N	1.36077558	-6.16106082	-0.00193940
C	-3.13358977	-2.68185913	-5.75342596
C	-5.08674135	-2.14948935	-4.30894679
C	2.98475831	-2.08491083	-5.39344116
C	4.71408306	-2.75703542	-3.73381907
C	1.66283274	-6.93855613	-1.19444228
C	1.54513931	-6.79591872	1.28997813
H	0.91337245	-7.69721648	1.37642426
H	2.59947281	-7.08857013	1.43906264
H	1.26174754	-6.08866405	2.08274894
H	1.07477626	-7.87208056	-1.20190181
H	1.39405673	-6.32676126	-2.06619096
H	2.73630035	-7.19481628	-1.23611467
H	-5.42749906	-1.31441553	-4.94698221
H	-5.63802088	-3.06363913	-4.59810292
H	-5.32711479	-1.91136382	-3.26578379
H	-3.34845363	-1.87225247	-6.47395642
H	-2.04733268	-2.80786892	-5.65384667
H	-3.58328469	-3.61886236	-6.12346162

```
  H     5.14121801  -3.54292747  -4.38072584
  H     5.29638758  -1.82935989  -3.86757110
  H     4.80558788  -3.06837679  -2.68293190
  H     3.34216322  -2.81710956  -6.13768604
  H     1.89232094  -1.99165855  -5.45714560
  H     3.44701451  -1.10528042  -5.60300483
```

[Yb$_2$HL$_2$(OH)(DMF)$_3$]$^-$-(V)

```
   146
 [Yb2HL2(OH)(DMF)3]--(V) complex optimized structure
  C     2.13685455  -2.46947402  -1.75810795
  C     2.89502329  -2.20236946  -2.93036451
  C     3.78610964  -3.14995390  -3.42925555
  C     3.92447925  -4.37432315  -2.78523056
  C     3.17152445  -4.68053758  -1.65658230
  C     2.28062349  -3.74265012  -1.14176020
  S     2.61719309  -0.69241793  -3.86737018
  C     3.75956267   0.41970704  -3.04132065
  C     5.00125640   0.64204987  -3.62187265
  C     5.88579645   1.54866566  -3.04232803
  C     5.53539762   2.23876925  -1.88135426
  C     4.29640537   2.02314846  -1.29676413
  C     3.33775869   1.10478903  -1.85195808
  S     3.83721454   3.05150280   0.10643331
  C     3.98596553   1.97785249   1.54739643
  C     5.22170090   1.85855981   2.16314234
  C     5.33538978   1.12460465   3.34382171
  C     4.21642676   0.50608772   3.90572241
  C     2.97586125   0.60503341   3.29500960
  C     2.78552023   1.38075426   2.09033074
  S     1.56508687  -0.25155735   4.02598219
  C     1.94868834  -1.90930033   3.43584857
  C     2.76109244  -2.73546652   4.19788970
  C     3.12740877  -4.00437630   3.73402007
  C     2.71005846  -4.45066035   2.48042408
  C     1.88085840  -3.64383120   1.71601805
  C     1.39590479  -2.36167138   2.17674790
  S     1.19826541  -4.30275060   0.19060020
 Yb   -0.97501920  -2.37352644   0.00790199
  O    -1.43248055  -4.00224575   1.67856438
  C    -1.44126498  -3.72053876   2.89623322
  N    -1.25886531  -4.61159631   3.88150037
  C    -0.98094378  -6.01381424   3.59322359
  N     3.94331516  -4.88099947   4.57455203
  O     4.24769723  -5.99376785   4.11741336
  O     0.50871999  -1.69745470   1.53380085
  N     6.63311284   1.02589132   4.01353009
  O     6.68329003   0.39079743   5.07694030
  O     1.62415623   1.54965943   1.56567199
 Yb    1.02306379   2.69828102  -0.27707559
  O     1.32640929   4.13087905  -1.72742333
  H     1.19818966   4.08727757  -2.69060420
  N     7.19190730   1.78714611  -3.66535294
  O     7.94315526   2.60721913  -3.12125852
  O     2.16478948   0.91589598  -1.31909247
  N     4.85718256  -5.38433153  -3.32948029
  O     5.49127159  -5.07923280  -4.34410113
  O     1.25766797  -1.58000762  -1.26141682
  S    -3.76659366  -3.27666630  -0.45834911
  C    -4.52310202  -2.16167400   0.74652805
  C    -3.68199858  -1.34240488   1.57005440
```

C	-4.32351616	-0.63699947	2.64695203
C	-5.70039188	-0.68316180	2.82192612
C	-6.48020212	-1.44912272	1.95520576
C	-5.89692517	-2.20697730	0.93944160
O	-2.40021065	-1.23537285	1.38832403
S	-3.29150159	0.10521415	3.92235554
C	-3.02497027	1.80891750	3.41565802
C	-3.34668705	2.79570465	4.34477046
C	-3.04566562	4.13019697	4.07818377
C	-2.53050303	4.51592357	2.84132266
C	-2.22405156	3.54538756	1.89614582
C	-2.37590863	2.15778464	2.19525133
N	-7.93678744	-1.50708583	2.15275919
O	-8.41231118	-0.84225581	3.08158115
N	-3.28969100	5.14747246	5.10533383
O	-2.82946893	6.28430014	4.90102368
S	-1.52251107	4.10385789	0.33921401
C	-2.53433004	3.30062967	-0.89963279
C	-3.84592120	3.69281767	-1.09021731
C	-4.58141478	3.11624086	-2.12807904
C	-3.99839146	2.18997796	-2.99156216
C	-2.67697267	1.80151618	-2.81698220
C	-1.87443079	2.32139792	-1.73444779
S	-1.96595581	0.62509256	-3.97368064
C	-3.04993900	-0.74869757	-3.61318993
C	-2.93325995	-1.37607101	-2.31238818
C	-3.89002673	-2.41965044	-2.03510627
C	-4.76609617	-2.89419990	-2.99878622
C	-4.75645044	-2.32310896	-4.27487579
C	-3.92540829	-1.23717205	-4.57243260
N	-5.63585021	-2.86540932	-5.31088974
O	-6.36925995	-3.81711123	-5.00254601
O	-2.00456216	-1.05932820	-1.48874680
N	-5.97049743	3.53495492	-2.34486372
O	-6.58009563	3.02776144	-3.29572774
O	-0.66794465	1.94815952	-1.52861455
O	-1.90237682	1.29330807	1.31917197
O	-0.92950083	-3.74086027	-1.93922197
C	-0.77174395	-3.39434711	-3.12847319
N	-1.07387987	-4.15529498	-4.19013168
C	-1.66848882	-5.47731036	-4.03020935
O	-8.58833026	-2.22116476	1.38050819
O	1.27058273	4.41896056	1.39382714
C	1.10755316	4.42311844	2.62653415
N	0.99222680	5.53836379	3.37021909
C	1.03679758	6.85732954	2.75050241
O	-6.44269191	4.37172336	-1.56222980
O	4.26559772	-4.46351146	5.69588554
O	7.45722080	1.15435493	-4.69681782
O	-3.91509083	4.81011236	6.11631801
O	-5.57889702	-2.35138618	-6.43810808
O	7.59936654	1.58851284	3.47781738
O	4.93008654	-6.46480419	-2.73498651
C	0.72792234	5.47290605	4.80143520
C	-1.22146903	-4.18612287	5.27484788
C	-0.89599461	-3.65163176	-5.54629773
H	4.36628816	-2.94960194	-4.33054590
H	3.28504101	-5.66059477	-1.19154897
H	6.24608335	2.94805850	-1.45475469
H	4.34636236	-0.05712405	4.83087394
H	6.11096779	2.33929900	1.75218029
H	3.03146293	-5.43592674	2.13816581
H	3.13439388	-2.41022491	5.17013049

H	5.29898776	0.12507556	-4.53477078
H	-5.45613961	-3.71472456	-2.79482291
H	-3.98709658	-0.78539953	-5.56391448
H	-6.18724214	-0.14877685	3.63869198
H	-2.38384552	5.57527954	2.62674399
H	-3.81924081	2.53681107	5.29317209
H	-4.60257317	1.78962973	-3.80682080
H	-4.32414056	4.44406344	-0.45988792
H	-6.53853818	-2.82942723	0.31356457
H	-2.05377783	0.30292025	1.55135814
H	1.54525554	-0.59484623	-1.45128931
H	1.03480658	3.48515560	3.21210608
H	-0.36111316	-2.40229728	-3.39817510
H	-1.60095669	-2.68301007	3.24646875
H	0.08339356	7.38588521	2.91777328
H	1.85681517	7.45309448	3.18502371
H	1.20573472	6.72336347	1.67354529
H	-0.25216346	5.92688990	5.02844099
H	0.71831012	4.42114879	5.12185353
H	1.51386860	6.00623972	5.36180808
H	-0.23175108	-4.31999278	-6.11836491
H	-1.86974965	-3.58449861	-6.05898444
H	-0.44703441	-2.64900416	-5.50909394
H	-1.71914331	-5.70350173	-2.95707388
H	-2.68425057	-5.48930809	-4.45883515
H	-1.05009188	-6.23258546	-4.54211655
H	-1.06475356	-6.16431757	2.50916988
H	0.03812497	-6.27386830	3.92456224
H	-1.70722603	-6.65686221	4.11679280
H	-0.22182643	-4.36861089	5.70314891
H	-1.44026820	-3.11063762	5.33496056
H	-1.97222096	-4.73829510	5.86385352

References

1. Wu, D.; Chen, A.; Johnson, C. An Improved Diffusion-Ordered Spectroscopy Experiment Incorporating Bipolar-Gradient Pulses. *J. Magn. Reson. Ser. A* **1995**, *115*, 260–264, https://doi.org/10.1006/jmra.1995.1176.
2. Sheldrick G.M. SADABS-2012/1. Bruker/Siemens Area Detector Absorption Correction Program. Bruker AXS Inc., Madison, Wisconsin, USA, 2012.
3. Sheldrick, G.M. M.: SHELXTL. Structure Determination Software Suite. Bruker AXS Inc: Madison, Wisconsin, USA 2000. *Sheldrick G.M.* SHELXTL, Structure Determination Software Suite, v. 6.1, Bruker AXS Inc., Madison: Wisconsin, USA, 2000.
4. APEX3. Crystallography Sotware Suite, Version 2018.7-2, BrukerAXS Inc., Madison, Wisconsin, USA 2016.
5. Farrugia, L.J. *WinGX*suite for small-molecule single-crystal crystallography. *J. Appl. Crystallogr.* **1999**, *32*, 837–838, https://doi.org/10.1107/s0021889899006020.
6. Macrae, C.F.; Sovago, I.; Cottrell, S.J.; Galek, P.T.A.; McCabe, P.; Pidcock, E.; Platings, M.; Shields, G.P.; Stevens, J.S.; Towler, M.; et al. *Mercury 4.0*: from visualization to analysis, design and prediction. *J. Appl. Crystallogr.* **2020**, *53*, 226–235, https://doi.org/10.1107/s1600576719014092.
7. Rigaku Oxford Diffraction, CrysAlisPro Software system, Version 1.171.42.51 2022, Rigaku Corporation, Oxford, UK, 2015.
8. Sheldrick, G.M. *SHELXT* – Integrated space-group and crystal-structure determination. *Acta Crystallogr., Sect. A: Found. Adv.* **2015**, *71*, 3–8. DOI: 10.1107/S2053273314026370
9. Palatinus, L.; Chapuis, G. *SUPERFLIP*– a computer program for the solution of crystal structures by charge flipping in arbitrary dimensions. *J. Appl. Crystallogr.* **2007**, *40*, 786–790, https://doi.org/10.1107/s0021889807029238.
10. Petříček, V.; Dušek, M.; Palatinus, L. Crystallographic Computing System JANA2006: General features. **2014**, *229*, 345–352, https://doi.org/10.1515/zkri-2014-1737.
11. Putz, H.; Brandenburg, K. Diamond - Crystal and molecular structure visualization, Crystal impact - H. Putz & K. Brandenburg GbR, Kreuzherrenstr. 102, D-53227 Bonn, Germany. https://www.crystalimpact.de/diamond.
12. Schomaker, V.; Trueblood, K.N. On the rigid-body motion of molecules in crystals. *Acta Crystallogr. Sect. B Struct. Crystallogr. Cryst. Chem.* **1968**, *24*, 63–76, https://doi.org/10.1107/s0567740868001718.
13. Lapaev, D.V.; Nikiforov, V.G.; Safiullin, G.M.; Galyaviev, I.G.; Dzabarov, V.I.; Knyazev, A.; Lobkov, V.S.; Galyametdinov, Y. Interligand energy transfer in europium(III) mesogenic adducts. *J. Struct. Chem.* **2009**, *50*, 775–781, https://doi.org/10.1007/s10947-009-0117-9.
14. Laikov, D.N. Fast evaluation of density functional exchange-correlation terms using the expansion of the electron density in auxiliary basis sets. *Chem. Phys. Lett.* **1997**, *281*, 151–156, https://doi.org/10.1016/s0009-2614(97)01206-2.
15. Perdew, J.P.; Burke, K.; Ernzerhof, M. Generalized gradient approximation made simple. *Phys. Rev. Lett.* **1996**, *77*, 3865. https://doi.org/10.1103/PhysRevLett.77.3865.

16. Laikov, D.N. A new class of atomic basis functions for accurate electronic structure calculations of molecules. *Chem. Phys. Lett.* **2005**, *416*, 116–120, https://doi.org/10.1016/j.cplett.2005.09.046.
17. Bakovets, V.V.; Masliy, A.N.; Kuznetsov, A.M. Formation Thermodynamics of Cucurbit[6]uril Macrocycle Molecules: A Theory Study. *J. Phys. Chem. B* **2008**, *112*, 12010–12013, https://doi.org/10.1021/jp802914g.
18. Podyachev, S.N.; Gubaidullin, A.T.; Syakaev, V.V.; Sudakova, S.N.; Masliy, A.N.; Saifina, A.F.; Burmakina, N..; Kuznetsov, A.M.; Shagidullin, R.R.; Avvakumova, L.V.; et al. Structural characterization and some coordinational aspects of tetrathiacalix[4]arenes functionalized by hydrazide groups. *J. Mol. Struct.* **2010**, *967*, 72–79, https://doi.org/10.1016/j.molstruc.2009.12.042.
19. Podyachev, S.N.; Masliy, A.N.; Semenov, V.E.; Syakaev, V.V.; Sudakova, S.N.; Voronina, J.K.; Ivanov, V.T.; Kuznetsov, A.M.; Gogolashvili, E.L.; Reznik, V.S.; et al. Silver mediated duplex-type complexes of pyrimidinophanes and their acyclic counterparts. *RSC Adv.* **2015**, *5*, 16017–16028, https://doi.org/10.1039/C4RA14070B.
20. Kovalenko, E.; Vilaseca, M.; Díaz-Lobo, M.; Masliy, A.N.; Vicent, C.; Fedin, V.P. Supramolecular Adducts of Cucurbit[7]uril and Amino Acids in the Gas Phase. *J. Am. Soc. Mass Spectrom.* **2015**, *27*, 265–276, https://doi.org/10.1007/s13361-015-1274-z.
21. Grishaeva, T.N.; Masliy, A.; Kuznetsov, A.M. Water structuring inside the cavities of cucurbit[n]urils (n = 5–8): a quantum-chemical forecast. *J. Incl. Phenom. Macrocycl. Chem.* **2017**, *89*, 299–313, https://doi.org/10.1007/s10847-017-0751-3.
22. Kovalenko, E.A.; Pashkina, E.A.; Kanazhevskaya, L.Y.; Masliy, A.N.; Kozlov, V.A. Chemical and biological properties of a supramolecular complex of tuftsin and cucurbit[7]uril. *Int. Immunopharmacol.* **2017**, *47*, 199–205, https://doi.org/10.1016/j.intimp.2017.03.032.
23. Gaussian 09, Revision E.01, M. J. Frisch, G. W. Trucks, H. B. Schlegel, G. E. Scuseria, M. A. Robb, J. R. Cheeseman, G. Scalmani, V. Barone, G. A. Petersson, H. Nakatsuji, X. Li, M. Caricato, A. Marenich, J. Bloino, B. G. Janesko, R. Gomperts, B. Mennucci, H. P. Hratchian, J. V. Ortiz, A. F. Izmaylov, J. L. Sonnenberg, D. Williams-Young, F. Ding, F. Lipparini, F. Egidi, J. Goings, B. Peng, A. Petrone, T. Henderson, D. Ranasinghe, V. G. Zakrzewski, J. Gao, N. Rega, G. Zheng, W. Liang, M. Hada, M. Ehara, K. Toyota, R. Fukuda, J. Hasegawa, M. Ishida, T. Nakajima, Y. Honda, O. Kitao, H. Nakai, T. Vreven, K. Throssell, J. A. Montgomery, Jr., J. E. Peralta, F. Ogliaro, M. Bearpark, J. J. Heyd, E. Brothers, K. N. Kudin, V. N. Staroverov, T. Keith, R. Kobayashi, J. Normand, K. Raghavachari, A. Rendell, J. C. Burant, S. S. Iyengar, J. Tomasi, M. Cossi, J. M. Millam, M. Klene, C. Adamo, R. Cammi, J. W. Ochterski, R. L. Martin, K. Morokuma, O. Farkas, J. B. Foresman, and D. J. Fox, Gaussian, Inc., Wallingford CT, 2016.
24. Cances, E.; Mennucci, B.; Tomasi, J. A new integral equation formalism for the polarizable continuum model: Theoretical background and applications to isotropic and anisotropic dielectrics. *J. Chem. Phys.* **1997**, *107*, 3032–3041, https://doi.org/10.1063/1.474659.
25. Cossi, M.; Barone, V.; Mennucci, B.; Tomasi, J. Ab initio study of ionic solutions by a polarizable continuum dielectric model. *Chem. Phys. Lett.* **1998**, *286*, 253–260, https://doi.org/10.1016/s0009-2614(98)00106-7.

26. Mennucci, B.; Tomasi, J. Continuum solvation models: A new approach to the problem of solute's charge distribution and cavity boundaries. *J. Chem. Phys.* **1997**, *106*, 5151–5158, https://doi.org/10.1063/1.473558.
27. Pollak, P.; Weigend, F. Segmented Contracted Error-Consistent Basis Sets of Double- and Triple-ζ Valence Quality for One- and Two-Component Relativistic All-Electron Calculations. *J. Chem. Theory Comput.* **2017**, *13*, 3696–3705, https://doi.org/10.1021/acs.jctc.7b00593.
